# The Homeodomain Iroquois Proteins Control Cell Cycle Progression and Regulate the Size of Developmental Fields

**DOI:** 10.1371/journal.pgen.1005463

**Published:** 2015-08-25

**Authors:** Natalia Barrios, Esther González-Pérez, Rosario Hernández, Sonsoles Campuzano

**Affiliations:** Department of Development and Differentiation, Centro de Biología Molecular Severo Ochoa, CSIC-UAM, Madrid, Spain; Harvard Medical School, UNITED STATES

## Abstract

During development, proper differentiation and final organ size rely on the control of territorial specification and cell proliferation. Although many regulators of these processes have been identified, how both are coordinated remains largely unknown. The homeodomain Iroquois/Irx proteins play a key, evolutionarily conserved, role in territorial specification. Here we show that in the imaginal discs, reduced function of *Iroquois* genes promotes cell proliferation by accelerating the G1 to S transition. Conversely, their increased expression causes cell-cycle arrest, down-regulating the activity of the Cyclin E/Cdk2 complex. We demonstrate that physical interaction of the Iroquois protein Caupolican with Cyclin E-containing protein complexes, through its IRO box and Cyclin-binding domains, underlies its activity in cell-cycle control. Thus, *Drosophila* Iroquois proteins are able to regulate cell-autonomously the growth of the territories they specify. Moreover, our results provide a molecular mechanism for a role of *Iroquois/Irx* genes as tumour suppressors.

## Author Summary

The correct development of body organs, with their characteristic size and shape, requires the coordination of cell division and cell differentiation. Here we show that the Iroquois proteins (Irx in vertebrates) slow down cell division in the *Drosophila* imaginal discs, in addition to their well-known role in cell fate and territorial specification. In humans, inactivating mutations at the *Irx* genes are associated to several types of cancer, thus allowing their classification as tumour suppressor genes. We have observed that *Drosophila* Iroquois genes similarly behave as tumour suppressor genes. Iroquois proteins belong to a family of homeodomain-containing transcriptional regulators. However, our results indicate that they control cell division by a transcription independent mechanism based on their physical interaction with Cyclin E containing complexes, a key player in cell-cycle progression. We have identified two evolutionary conserved domains of Iroquois proteins, different from the homeodomain, involved in that interaction. This new function of Iroquois proteins places them in a key position to coordinate growth and differentiation during normal development. Our results further suggest a molecular mechanism for their role in tumour suppression. Future studies of Irx genes should help to determine if a similar mechanism could operate to help cancer progression when Irx activity is compromised.

## Introduction

Development of the different body parts in multicellular organisms is a stepwise process that entails the specification within developmental fields of territories with the ability to acquire different fates. Morphogens, which orchestrate such territorial specification, are also able to regulate territorial growth [1]. There is increasing evidence that, conversely, the regulation of the size of the developmental fields over which morphogens spread and operate is paramount for territorial specification [2–4]. For instance, in two paradigms of morphogenetic fields—the vertebrate limb primordium and the *Drosophila* imaginal discs- two sources of morphogens are present at opposite sites. Since activity of one of them is prevented by the action of the other one, the morphogenetic field must reach a critical size for that morphogen to escape from inhibition and be able to initiate the territorial specification program [5–8]. Therefore, the identification of the genes that control cell proliferation in developmental fields is key to a better understanding of how cell proliferation and territorial specification are coordinated during development.

Here we address the role of the *Drosophila* Iroquois Complex genes (*Iro* genes) in cell proliferation. The three *Iro* genes, *araucan* (*ara*), *caupolican* (*caup*) and *mirror* (*mirr*), encode highly related and evolutionarily conserved homeodomain transcription factors of the TALE family [9–11]. They play key roles in development that range from territorial specification to pattern formation (reviewed in [12]). Namely, at the early second larval instar, *Iro* genes are expressed in sub-regions of the wing and eye imaginal discs where they define the prospective notum and the dorsal compartment of the eye, respectively [13–15]. *Iro* genes also contribute to the growth of the discs by generating organising borders at the confrontation of *Iro*-expressing and non-expressing cells [13–15]. In the dorsal compartment of the eye disc, Iro proteins repress the expression of *fringe* (*fng*), thus restricting the activation of the Notch pathway at the dorso/ventral (D/V) compartment border. This triggers growth of the entire eye disc and the initiation of retinal differentiation from its posterior rim [14, 16, 17], reviewed in [18]. Moreover, Iro proteins may also have a more direct role in the control of cell proliferation. Thus, clones of *iro* ^*-*^ cells in the eye disc are larger than the control ones [13, 19] and, conversely, generalized over-expression of *ara* in the wing disc reduces wing size [9]. Furthermore, vertebrate *Irx* genes (orthologs of *Drosophila Iroquois* genes) appear to function as tumour suppressor genes (TSG) for certain types of cancer [20–23].

In this work we show that Iro proteins indeed control cell proliferation, both during normal development and in several established *Drosophila* tumour-like models. Iro proteins specifically regulate the G1-S transition of the cell cycle by modulating the activity of the CyclinE/ Cyclin dependent kinase 2 (CycE/Cdk2) complex. Unexpectedly for transcription factors, they are able to do so by a non-transcriptional mechanism. Thus, we demonstrate that Caup forms a protein complex with CycE in S2 cells and disclose the function of the evolutionarily-conserved IRO·box domain of Caup for that physical interaction and for cell cycle regulation *in vivo*. Our results support a direct, cell-autonomous role of *Drosophila Iro* genes in the regulation of cell cycle progression. This function of the Iro genes uncovers a new layer of regulation of organ size during development and may account for their behaviour as tumour suppressor genes.

## Results

### Loss of function of *Iro* genes enhances cell proliferation

**Fig 1:**
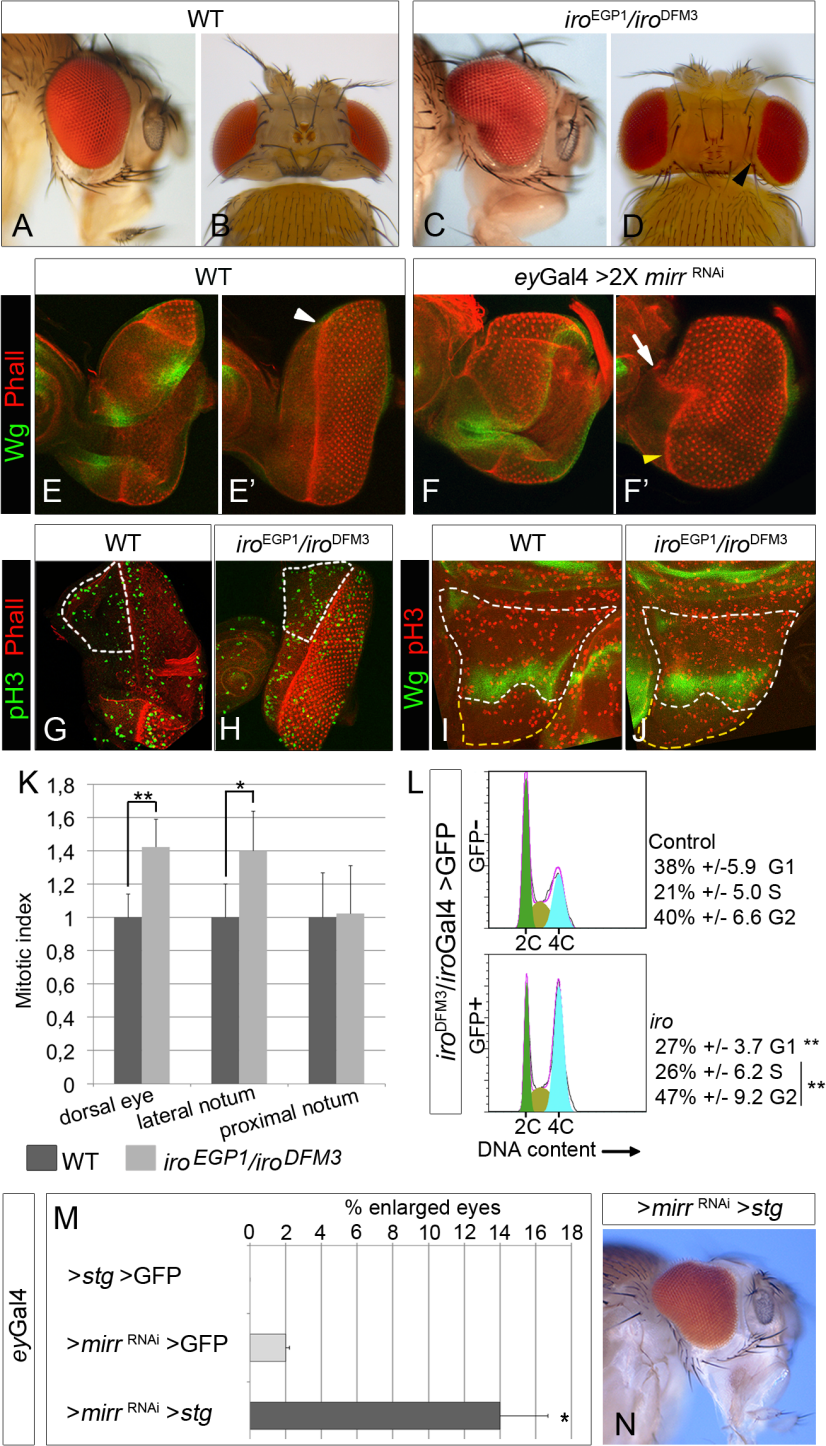
Cell-autonomous increase in cell proliferation in *iro* mutants. Lateral (A, C) and dorsal (B, D) views of heads of flies of the indicated genotypes. (E- F’) Expression of Wg (green) and Phalloidin staining (red) in wild-type (E, E’) and *ey*Gal4*>mirr* RNAi (two copies of *mirr* RNAi, flies raised at 29°C, F, F’) eye discs. (E and E’ and F and F’ are different focal planes of the same disc). Arrowheads and arrow mark the position of the morphogenetic furrow. (G-K) Mitotic patterns (phospho-Histone H3 staining, green, G, H; red I, J) and quantification of the relative mitotic index (K) in *Iro*-expressing territories (white dotted areas in G-J) and in the prospective proximal notum (yellow dotted areas in I, J). (*p<0.05; **p<0.005). (L) G1/S transition is accelerated in *iro* mutant cells. Representative profiles of FACS analysis of cells dissociated from *iro* ^DFM3^/*iro*Gal4 UAS-GFP wing discs. (The differences in the percentages of G1 and (G2+S) cells between the GFP^+^ and GFP^-^ populations are statistically significant, **p<0.005). (M, N) Reduction of Mirr levels (one copy of UAS-*mirr* RNAi, larvae raised at 25°C) and over-expression of *stg* synergistically interact to increase eye size. (M) Quantification of the fraction of enlarged eyes in flies of the indicated genotypes (average from two independent experiments, n>100, *p<0.05). (N) Representative mutant enlarged eye. In this and following figures, the eye discs are oriented dorsal up and posterior to the right, and the wing discs, ventral up and posterior to the right. Quantitative data are shown as arithmetic mean +/- SD (error bars). WT, wild-type.

We found that *iro* ^EGP1^ homozygous flies and those harbouring the *iro* ^EGP1^ allele combined with a deficiency of the whole Iro-C (*iro* ^DFM3^, S1A Fig) had dorsally enlarged eyes (Fig 1A–1D, 5% of *iro* ^EGP1^ flies, 36% of the *iro* ^EGP1^ /*iro* ^DFM3^ everted flies). The cephalic capsule was morphologically normal, except for alterations in the number of orbital bristles (Fig 1D, arrowhead). In third instar wild-type eye imaginal discs, the three *Iro* genes are expressed in a dorsal domain ahead of the morphogenetic furrow (S1B and S1C Fig, see also [10, 14]). In contrast, in *iro* ^EGP1^ /*iro* ^DFM3^ eye discs the expression of *caup* was undetectable and that of *ara* was strongly decreased, while *mirr* expression was not affected (S1D–S1F Fig). Dorsally enlarged eyes were also found in 51% of the flies depleted of Mirr (by expression of two copies of UAS-*mirr* RNAi driven by *ey*Gal4 at 25°C).

Ectopic D/V organisers, induced by clones of *iro* mutant cells in the dorsal compartment of the eye disc, can promote dorsally enlarged eyes [14, 19]. In adult eyes, the D/V organizer is visualized as the symmetry axis of the ommatidia field, named the equator (S1G and S1G’ Fig; [24]). However, ectopic equators were not found in retina sections of adult enlarged *iro* ^EGP1^ */iro* ^DFM3^ eyes (S1H and S1H’ Fig). Enlarged eyes have also been associated with reduced activity of the Wingless (Wg) pathway, which allows morphogenetic furrow initiation from the lateral margins of the disc [25]. While similar advance of the morphogenetic furrow was found in the dorsal domain in Mirr depleted eye discs (*ey-Gal4> 2 X UAS-mirr* RNAi, Fig 1F’, arrow), expression of *wg* was not apparently modified (Fig 1E and 1F, see also [14, 19]). Thus, we can rule out the generation of ectopic D/V organisers or insufficiency for Wg as the cause(s) of the observed eye enlargements.

Next, we monitored the rate of cell proliferation and the occurrence of cell death in *iro* ^EGP1^ */iro* ^DFM3^ eye discs, as their modifications might explain the enlarged eyes. Indeed the mitotic index was significantly increased in the *Iro* expressing domain, as compared to similar regions of wild-type discs (Fig 1G, 1H and 1K). This increase was not specific of the eye disc, since it also occurred in the lateral-notum region of *iro* ^EGP1^ */iro* ^DFM3^ wing discs (Fig 1I–1K, the lateral notum is delimited proximally by *wg* expression and distally by the most proximal of the wing hinge folds). Notably, the mitotic index was not altered in the region of *iro* ^EGP1^ */iro* ^DFM3^ wing discs proximal to the domain of *wg* expression (a region where *Iro* genes are not expressed at the third instar [12]), when compared to that of a similar region of wild-type discs (Fig 1I–1K).

We analyzed the cell cycle profiles of *iro* mutant cells using *iro* ^DFM3^/*iro*Gal4 UAS-GFP wing imaginal discs, which express GFP in the *ara/caup* domain [26]. *iro*Gal4 is a hypomorphic *iro* allele [26] and *iro* ^DFM3^ is a null allele (S1A Fig). We separated the GFP^+^ and GFP^-^ cell populations by FACS. In wild type wing discs, the cell cycle profile of wing pouch disc cells (mostly *Iro* non-expressing cells) and that of the rest of the disc (most of them *Iro*-expressing cells) are very similar [27]. Thus, GFP^-^ cells represented the internal control. Indeed, their cell cycle profile (38% in G1, 21% in S and 40% in G2, Fig 1L) was very similar to that previously described for wild-type cells from whole wing discs [28] and for wing pouch disc cells [27]. However, *iro* mutant GFP^+^ cells showed a cell cycle profile statistically different from that of rest of the wing disc cells (27% in G1, 26% in S and 47% in G2, Fig 1L). These alterations in the cell cycle profile resembled those caused by over-expression of *cycE* [28] and suggested that reduced levels of Iro proteins accelerate the passage through the G1 phase. In sum, these results allow us to conclude that Iro proteins cell-autonomously restrict cell proliferation.

We reasoned that an increase in the rate of the G2-M transition in the *iro* mutant eye discs should enhance eye overgrowth. Indeed, we found a synergistic effect on dorsal eye growth when *string* (*stg*), a phosphatase that drives the G2-M transition [29], was expressed in a background of slightly reduced expression of *mirr* (Fig 1M and 1N). We conclude that the reduced levels of Iro proteins in the dorsal territory of the *iro* eye disc induced over proliferation that resulted in dorsal eye overgrowth.

We also found an increased number of apoptotic cells in the *iro* territories of the mutant discs (S1M–S1P’ Fig). This increased apoptosis might help compensate the excess of proliferation, and reduce the extent of overgrowth especially in the notum (that was only slightly deformed in *iro* ^EGP1^ mutants, S1I–S1L Fig). It further precludes precise analysis of the doubling time of *iro* mutant cells.

### Over-expression of *Iro* genes restricts cell proliferation

**Fig 2:**
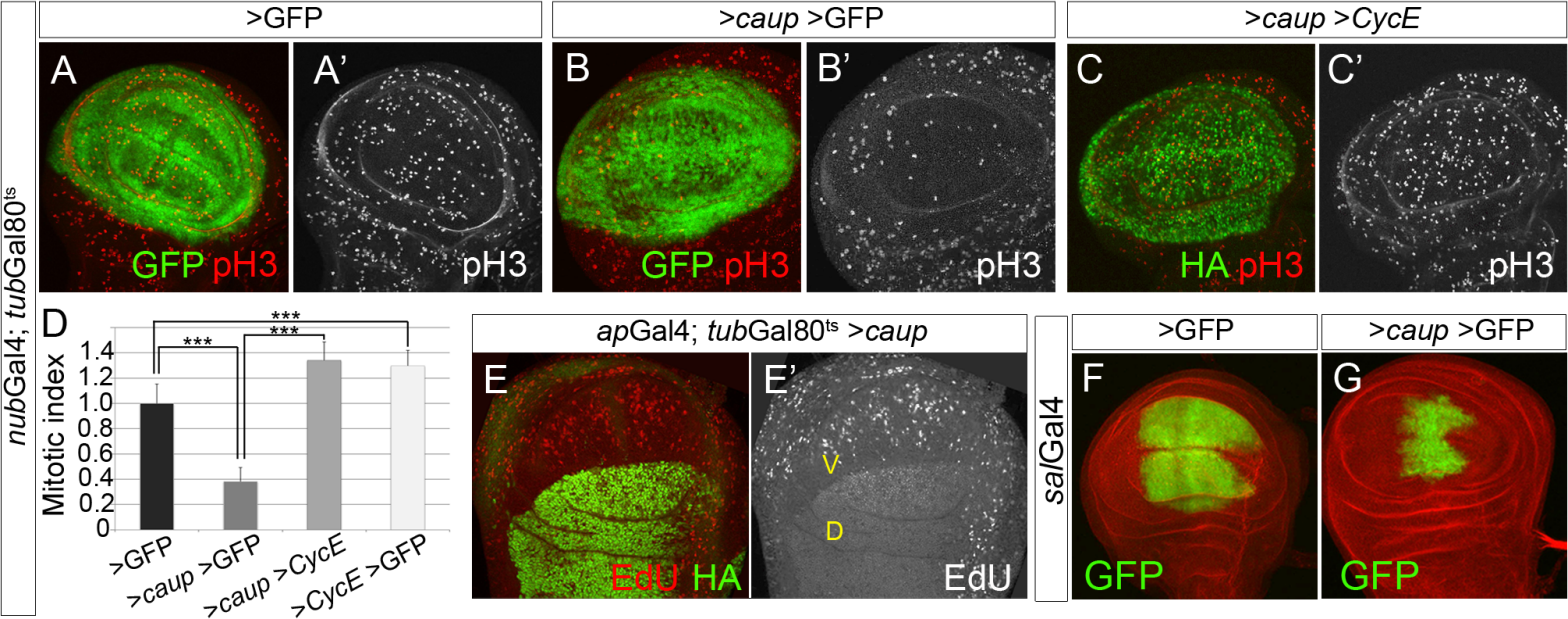
Over-expression of *caup* inhibits cell cycle progression. (A-C’) Mitotic pattern (pH3 staining) of wing imaginal discs expressing the indicated transgenes driven by *nub*Gal4 during 16 h prior to dissection (expression domain shown in green). (D) Quantification of the relative mitotic index in the *nub* territory in the indicated genetic backgrounds (***p<0.00001). (E, E’) Pattern of S phase cells (assayed by EdU incorporation) in wing discs expressing *caup*-HA (green in E) in the dorsal (D) compartment (*ap*Gal4 driver). Compare the pattern of EdU incorporation in the dorsal and control ventral (V) compartment. (F, G) Over-expression of *caup* driven by *sal*Gal4 reduces the size of the *sal* territory (labelled by GFP, disc counterstained with phalloidin, red).

Next, we tested whether over-expression of *Iro* genes caused the opposite effect to their loss of activity, that is, a reduction of cell proliferation. Since generalized expression of any of the *Iro* genes in the eye disc eliminates the D/V organiser and prevents growth of the eye disc and eye formation [14, 16, 17], we examined the effect of *caup* excess of function in the wing disc. We over-expressed *caup*-HA (henceforth *caup*) either in its normal expression domain, the prospective notum (using the *ap*Gal4 driver) or in the wing pouch (*nub*Gal4 driver). We assayed the effect of transient over-expressions of *caup* using of the Gal4/Gal80^ts^ system. We combined the *nub*Gal4 and *ap*Gal4lines with a *tub*Gal80^ts^ transgene [30]. At 17°C, (permissive temperature for Gal80^ts^), Gal80 inhibits Gal4 activity. *nub*Gal4 (or *ap*Gal4); *tub*Gal80^ts^; UAS-*caup* larvae were raised at 17°C, and transferred to 29°C (to inactivate Gal80^ts^) 16 hours prior to their dissection at late third larval instar. Both in the *nub* and *ap* domains, *caup* over-expression caused a significant reduction in the mitotic index (Fig 2A–2B’ and 2D; S2J and S2K Fig), Similar reduction in the mitotic index also occurred upon forced expression of *ara* or *mirr* (S2D, S2E and S2H Fig). We also observed a decreased incorporation of the thymidine analogue EdU in the cells over-expressing *caup* (Fig 2E and 2E’). Cell size was not noticeably affected by transient *caup* over expression (S2A and S2B Fig). Since it also was unmodified by depletion of CycE in similar experimental conditions (S2C Fig), we assume this could be due to the transient over-expression of the transgenes.

**Fig 3:**
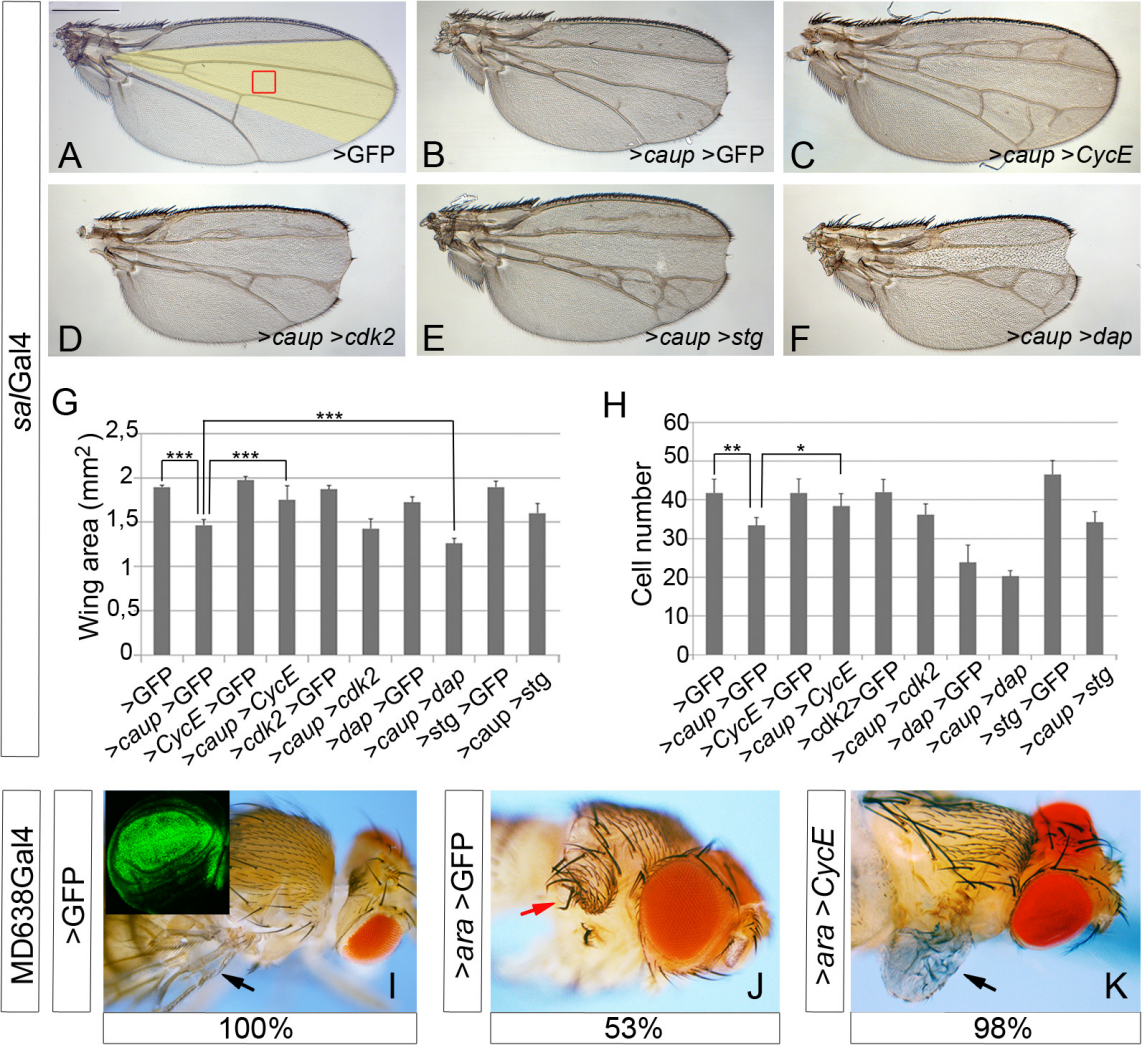
Genetic interactions of *caup* with cell cycle regulators. (A-F) Representative wings from flies of the indicated genotypes. Scale bar in A represents 500 μm. The region of the adult wing derived from the *sal*-expressing domain is shown in yellow in A. (G, H) Quantification of wing area (G, n = 10) and of the number of cells in a fixed wing area, similar to the region boxed in A (H, n = 5, calculated from the number of trichomes) for the indicated genotypes. ***p<0.0001; **p<0.005; *p<0.05. (I-K) Restoring cell proliferation by exogenously provided CycE recovers wing development in flies over-expressing *ara* (arrows). Transgene expression was driven by MD638Gal4 (expression domain in green in the inset in I). Red arrow in J indicates the notum-like structure that develops after *ara* over-expression (53% of the cases). The remaining MD638Gal4>*ara>*GFP flies present a wing stump and do not develop extra notum tissue. 98% of flies co-expressing *ara* and *cycE* show partially recovered wings (black arrow in K) and never develop a double notum (n>90).

To analyze the effect of much prolonged over-expression of *caup* in the wing disc, compatible with the development of the adult wing, we resorted to the *sal*Gal4 line. This Gal4 line drives expression of UAS genes in the central wing pouch of the wing disc from early third instar until 4h of pupal development (Fig 2F, [31]). Accordingly to a decreased cell proliferation in the *sal* domain of the wing discs caused by *caup* over-expression (S2M–S2M” Fig), we found a significant reduction in the size of this domain (Fig 2G, compare with F;) and of the adult wings (Fig 3A, 3B and 3G and S3A Fig). Furthermore, the mutant wings showed altered venation pattern and wing margin notches (Fig 3A and 3B). Wing notches were also found associated to cell cycle arrest caused by depletion of CycE (S3D Fig) and by the over-expression of *dacapo* (*dap*), ortholog of the Cyclin-dependent kinase (Cdk) inhibitor p21 [32], (S3E Fig) and could be attributed to reduced *wg* expression at the prospective wing margin in the *sal*Gal4>*caup* wing discs (S3B–S3C’ Fig). In addition, *sal*Gal4>*caup* wings showed enlarged cells in the *sal* domain (Fig 3H; S3J and S3K Fig). Small wings with vein patterning defects also result from over-expressing *ara* [9].

Although some cells entered apoptosis after *caup* over-expression (S4E and S4E’ Fig), their contribution to the mutant phenotype was apparently minimal. Co-expression of *caup* with the apoptosis inhibitor DIAP1 [33], reduced apoptosis (S4E–S4F’ Fig) but did not modify either the size, vein pattern or notches of wings over-expressing *caup* (S4A, S4B and S4I Fig). *sal*Gal4 driven expression lasts until 4 h after puparium formation [31]. Thus, to rule out the possibility that cell death during pupal stages could contribute to the mutant phenotype of *sal*Gal4>*caup* flies, we over-expressed *caup* in heterozygous conditions for *Df(3L)H99*. This deficiency removes the apoptosis inducing genes *reaper*, *hid* and *grim* [34] and halving the copy number of these genes largely reduces induced cell death [35]. We found that such reduction of apoptotic-inducing proteins did not modify the wing phenotype of *sal*Gal4>*caup* flies (S4C, S4D and S4I Fig).

These results indicate that elevated levels of Iro proteins restrict cell proliferation in the wing imaginal discs.

### *caup* genetically interacts with *CycE*

To further analyze the role of Iro proteins on cell cycle progression we searched for genetic interactions between *caup* and several cell cycle regulators. Co-expression of *caup* (*sal*Gal4 driver) with the G2-M regulator *stg* [32], which on its own only slightly decreased cell size (Fig 3H; S3F Fig), did not rescue the effects of *caup* over-expression (Fig 3B, 3E, 3G and 3H).

Next we investigated the interaction of *caup* with G1/S regulators. CycE binds to and activates Cdk2 to drive the G1-S transition [32]. While over-expression of *CycE* or *Cdk2* (*sal*Gal4 driver) did not modify wing or cell size (Fig 3G and 3H; S3G and S3H Fig), the co-expression of *caup* with *CycE* reverted all aspects of the *caup* over-expression adult phenotype (Fig 3A–3C, 3G and 3H; S3J–S3L Fig). Nevertheless, no reversion of the phenotype was observed by co-expressing *cdk2* (Fig 3D, 3G and 3H). In contrast, the Cdk inhibitor *dap* [32], whose over-expression reduced wing size and cell number and caused wing notches (Fig 3G and 3H; S3E and S3I Fig), enhanced the *caup* over-expression effect (Fig 3B and 3F–3H).

These results suggested that CycE, but not Cdk2, becomes a limiting factor for cell proliferation in the presence of high levels of Caup. Therefore, we examined if exogenously provided CycE could recover cell proliferation in cells over-expressing *caup* (*nub*Gal4 and *ap*Gal4 drivers). Fig 2A–2D and S2J–S2L Fig showed that this was indeed the case. Similar interactions were observed between *ara* or *mirr* and *CycE* (S2D–S2I Fig). Conversely, we found that co-expression with the F-box protein Archipelago (Ago), which induces CycE degradation through the proteosome pathway [36], significantly reduced the size of the *sal*Gal4>*caup* wings (S5A–S5D and S5G Fig). However, depletion of Ago (by expression of *ago* RNAi), which increased wing size (S5E and S5G Fig) did not recover but even enhanced the *caup*-over expression phenotype (S5B, S5F and S5G Fig). This effect could be attributed to the stabilization of unknown targets of Ago (other than CycE) by the depletion of this protein.

Next, we wonder whether similar insufficiency for CycE and the resulting impaired cell proliferation, could underlie other adult phenotypes caused by *Iro* genes over-expression. Associated to ectopic expression of *ara* in the prospective wing pouch, wings are absent and extra notum tissue develops ([37–39] and Fig 3J). Interestingly, co-expression of *ara* and *CycE*, which restored cell proliferation, allowed differentiation of a wing, albeit of a reduced size (Fig 3K). These results agree with those of [8], which showed that decreased cell proliferation in the wing pouch from early larval stages causes wing loss and duplication of body wall structures. In sum, these genetic interactions further support the regulation of cell cycle progression by Iro proteins at the G1-S transition suggested by the cell cycle profile analyses.

### *caup* over-expression inhibited the activity of the CycE/Cdk2 complex

**Fig 4:**
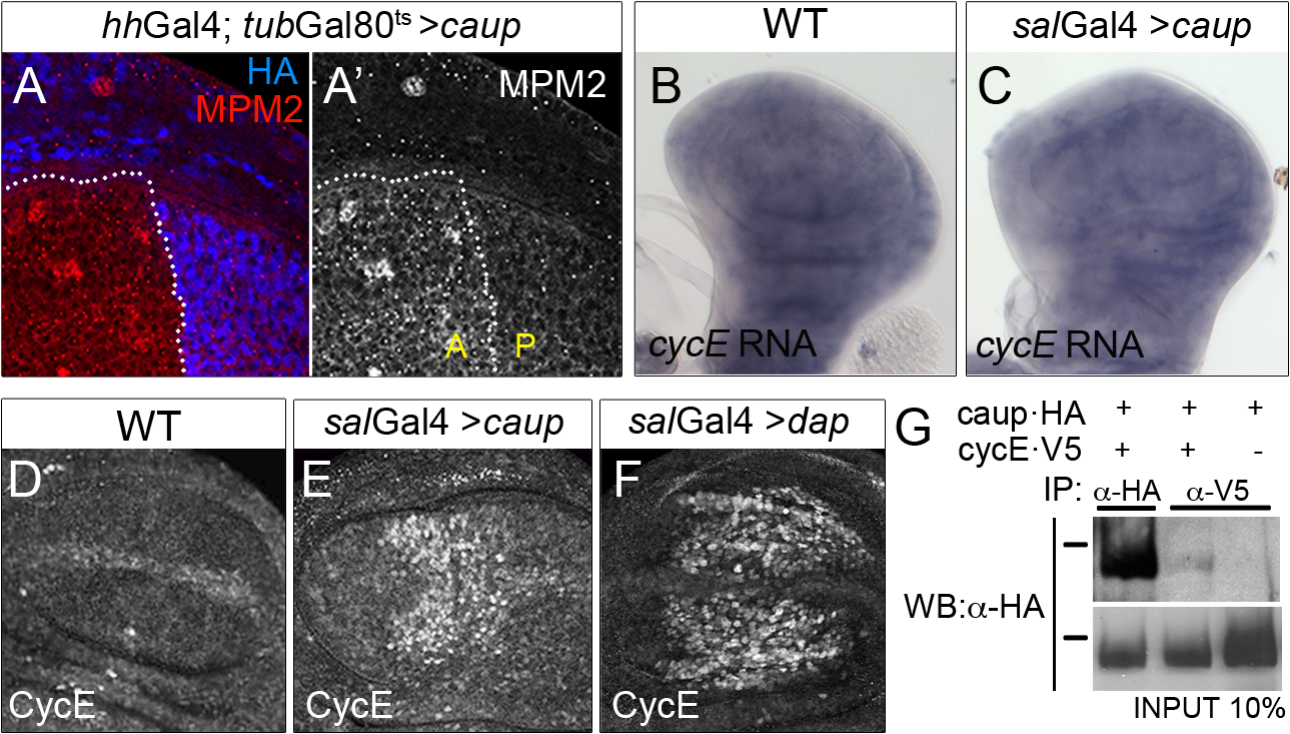
Functional and physical interaction of Caup with the CycE/Cdk2 complex. Activity of the CycE/Cdk2 complex, monitored by MPM-2 staining (A, A’); *cycE* transcription (detected by *in situ* hybridization, B, C) and CycE accumulation (detected by immunostaining, D-F) in wing imaginal discs of the indicated genotypes. (G) Caup co-immunoprecipitates with CycE in S2 cells. Western blot of protein extracts from S2 cells expressing the indicated tagged proteins, immunoprecipitated with anti-HA or anti-V5 antibodies and probed with anti-HA. Black bars indicate position of the 100 KDa protein marker.

In *Drosophila*, the activity of the CycE/Cdk2 complex is required and sufficient for G1-S transition [32]. We examined the activity of this complex in cells that ectopically express *caup* by MPM-2 staining. This antibody recognizes a CycE/Cdk2 regulated protein complex that assembles into the histone locus body and is visualized as nuclear dots [40]. As shown in Fig 4A and 4A’, *caup* over-expressing cells of the posterior compartment (*hh*Gal4 driver) displayed lower punctuated staining than control anterior cells indicating a decreased activity of the CycE/Cdk2 complex.

As we have shown above, CycE is a limiting component in *caup* over-expressing cells. Thus, the decreased activity of the CycE/Cdk2 complex could result from repression of *CycE* expression. However, transcription of *CycE* in the wing disc was not noticeably modified by *caup* forced expression (Fig 4B and 4C, see also S6A–S6C Fig). Interestingly, CycE protein levels were strongly increased (Fig 4D and 4E), even when apoptosis was reduced in *sal*Gal4>*caup* discs (S4G–S4H’ and S4J Fig). This suggested the stabilization of CycE protein when *caup* was over-expressed. Since phosphorylation of CycE by the CycE/Cdk2 complex is essential for its degradation [41], this result also supported that Caup reduced the activity of the CycE/Cdk2 complex. Similar increase in CycE levels was found associated to the inhibition of the CycE/Cdk2 complex by *dap* over expression (Fig 4F). Since mRNA and protein levels of *dap* were not modified in *caup* over-expressing cells (S6D–S6I Fig), the decreased activity of the CycE/Cdk2 complex in *caup* over expressing cells cannot be attributed to a deficiency of CycE or to excess amount of Dap.

### Caup bind to a CycE-containing protein complex

Putative Cyclin-binding sites have been identified in the three *Drosophila* Iro proteins (Eukaryotic Linear Motiv server, http://elm.eu.org). Hence, we wondered whether the reduction of CycE function in *caup* over-expressing cells (despite their higher than normal CycE levels) might be due physical interaction of Caup with CycE containing complexes. We tested for this interaction by co-immunoprecipitation of Caup-HA and CycE-V5 from *Drosophila* S2 cells. As shown in Fig 4G, Caup-HA was present in CycE-containing complexes.

**Fig 5:**
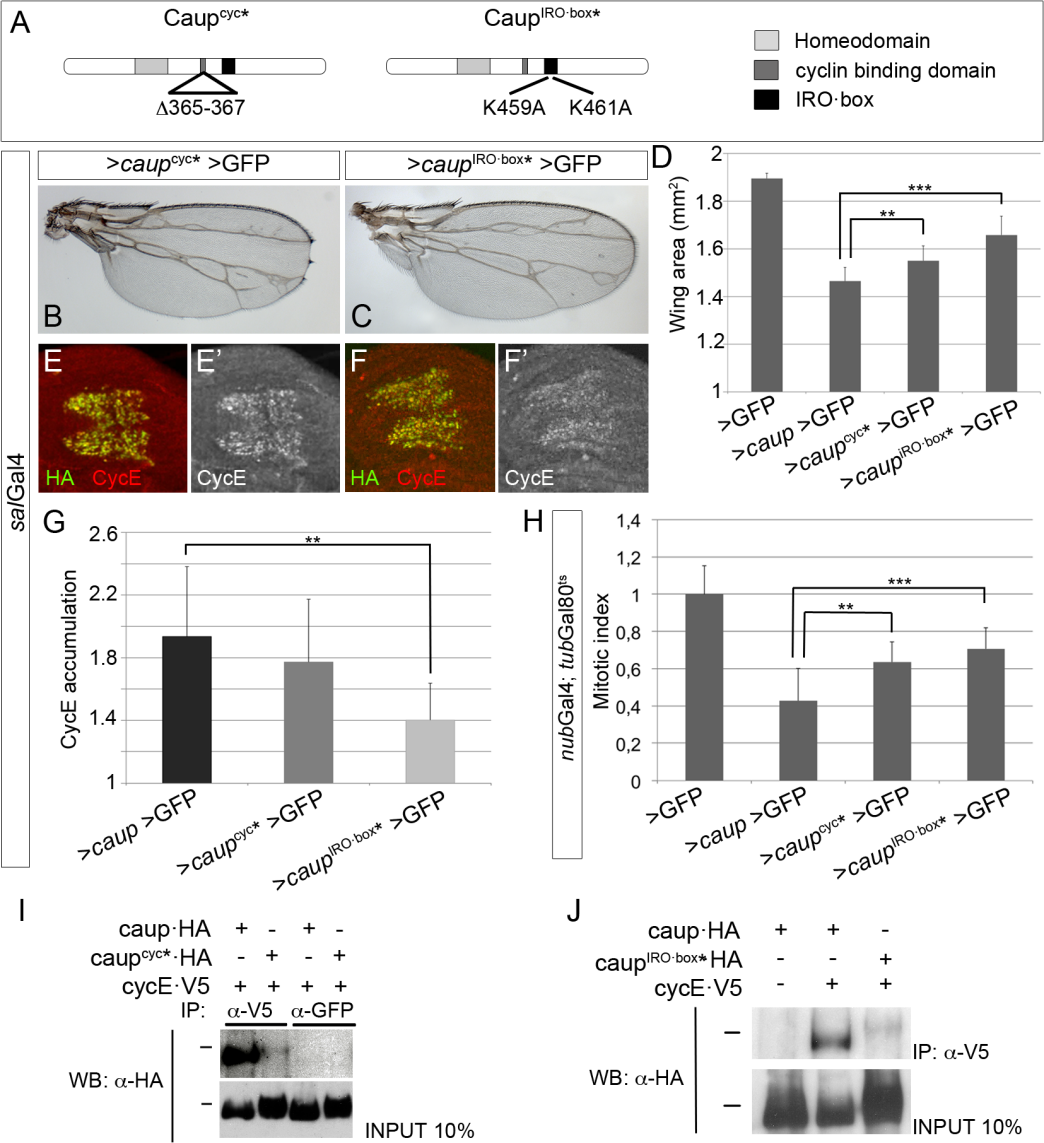
Structure-function analysis of Caup. (A) Domain structure of Caup. The amino acid changed in the Cyc-binding domain and IRO·box in the novel mutants are indicated. (B, D) Representative wing phenotypes associated to Caup^cyc^* and Caup^IRO·box^* over-expression (B, C) and quantification of wing sizes of flies over-expressing the indicated transgenes (D). (E-G) Accumulation of CycE in wing imaginal cells that ectopically express Caup^cyc^* or Caup^IRO·box^*, quantified in G. (B-G, over-expression driven by *sal*Gal4). (H) Mitotic index in the *nub* territory of wing discs over-expressing the indicated transgenes. In all cases, quantifications are shown in relation to those performed in *sal*Gal4>*caup*>*GFP* or *sal*Gal4>GFP control wing discs and wings from larvae reared in parallel to the experimental ones. (I, J) Interaction of the different Caup proteins with CycE-containing complexes. Western blots of protein extracts from S2 cells expressing CycE-V5 and the different Caup-HA proteins, immunoprecipitated with the indicated antibodies and probed with anti-HA. Black bars indicate the position of the 100 kDa protein marker.

Next, we tested whether the putative Cyc-binding site present in Caup mediated the interaction with CycE and, therefore, its effect on cell cycle regulation. We mutated this site and over-expressed the resulting protein (Caup^cyc^*, Fig 5A) in wing discs. Caup^cyc^* was less effective than wild-type Caup in reducing wing size (Fig 5B and 5D) and in repressing cell proliferation (Fig 5H), although it appeared similarly effective than wild-type Caup in inducing CycE accumulation (a read-out of the inhibition of CycE/Cdk2 complex activity, Fig 5E, 5E’ and 5G). In agreement with our working hypothesis, the decreased ability of Caup^cyc^* to reduce wing size and cell proliferation was paralleled by its compromised ability to co-immunoprecipitate with CycE in S2 cells (Fig 5I).

These results suggest that Caup may be interacting with CycE-containing complexes through additional domain(s). Iro/Irx proteins harbour a conserved stretch of 14 amino acids, the IRO·box, whose function is unknown [11]. We mutagenized it changing its two conserved positively charged amino acids into Alanine (Caup^IRO·box^*, Fig 5A) and assayed its activity *in vivo* and its ability to interact with CycE-containing complexes as described for Caup^cyc^*. Caup^IRO·box^* was much less effective than wild-type Caup and Caup^cyc^* in interfering with cell cycle progression as shown by its effect on the mitotic index (Fig 5H), wing size (Fig 5C and 5D) and CycE accumulation (Fig 5F and 5G). Accordingly, Caup^IRO·box^* showed a highly reduced ability to co-immunoprecipitate with CycE (Fig 5J). Since Caup^cyc^* and Caup^IRO·box^* were still able to repress cell proliferation, albeit less than wild-type Caup, we generated a double mutant *caup* ^cyc^*^- IRO·box^*. It still reduced wing size when over-expressed to a similar extent than Caup^IRO·box^* (S7D and S7E Fig).

**Fig 6:**
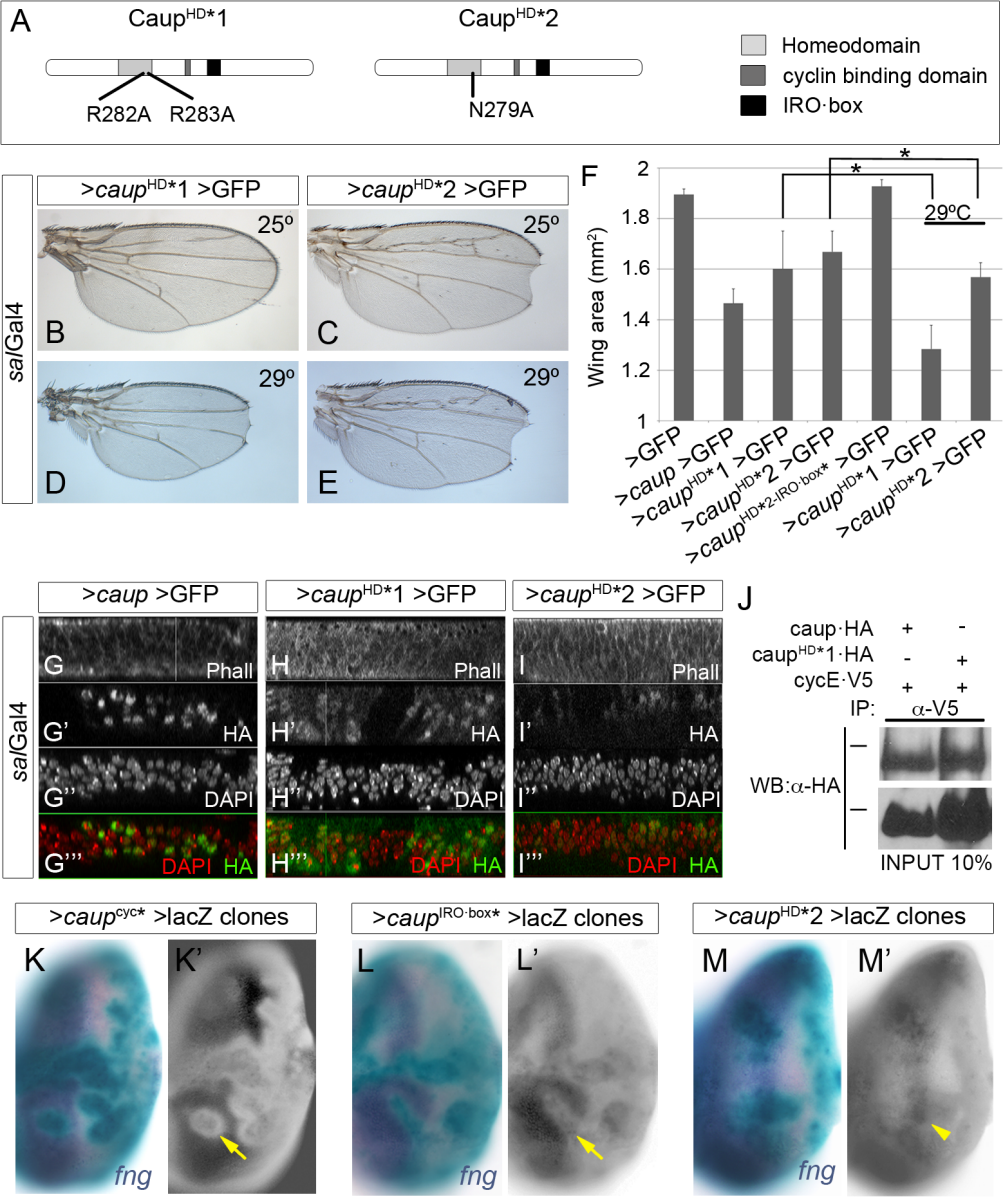
Functional analysis of homeodomain-mutant Caup proteins. (A) Domain structure of Caup. The position of the point mutations generated in the homeodomain of Caup^HD^*1 and Caup^HD^*2 proteins is indicated. (B-E) Representative wing phenotypes associated to the over-expression of *caup* ^HD^*1 or *caup* ^HD^*2 at the indicated temperatures. (F) Wing areas of flies expressing the indicated transgenes driven by *sal*Gal4 at 25°C, save when otherwise indicated. (G-I”‘) Sub-cellular localization of the different Caup proteins. Wild-type Caup localized to the cell nuclei (G-G”‘). Caup^HD^*1 (H-H”‘) and Caup^HD^*2 (I-I”‘) are also found diffusely distributed in the cytosol. H- I”‘ images were taken with higher laser intensity than G-G”‘ because Caup^HD^*1 and Caup^HD^*2 accumulate at lower levels than wild-type Caup. (J) Interaction of Caup^HD^*1 with CycE-containing complexes. Western blots of protein extracts from S2 cells expressing CycE-V5 and the indicated Caup-HA proteins, immunoprecipitated with anti-V5 antibody and probed with anti-HA. Black bars indicate the position of the 100 kDa protein marker. (K-M´) Transcriptional activity of different Caup* proteins. Clones of cells expressing caup* and *lacZ* are marked by X-Gal staining (green). *fng* mRNA (*in situ* hybridization) is shown in blue (K, L and M) and separately in K’, L’ and M’. Caup^cyc^* (K, K’) and Caup^IRO·box^* (L, L’) cell- autonomously repress *fng* expression (arrows) (The apparent decrease in *fng* expression around the clones over expressing Caup^cyc^* or Caup^IRObox^* is due to the epithelial folds that surround them, as previously shown for *caup* over-expressing clones [60]). Caup^HD^*2 does not repress *fng* expression (arrowhead; M, M´).

The functional differences observed between wild-type Caup, Caup^cyc^* and Caup^IRO·box^* could not be attributed to an altered sub-cellular localization, significantly different levels of expression or stability, since these were similar (S7A–S7C and S7F Fig). Both Caup^cyc^* and Caup^IRO·box^* retained the ability to act as transcriptional regulators (monitored by repression of *fng*, a direct target of *Iro* genes [42], Fig 6K–6L’) and accordingly, over-expression of Caup^IRO·box^* in the eye disc prevented eye development (S7I Fig). Thus, these results suggest that Caup inhibits the activity of the CycE/Cdk2 complex by physical interaction mediated, at least in part, by both the Cyc-binding domain and the IRO·box rather than by a transcriptional-dependent mechanism.

To further support this conclusion, we generated additional Caup mutants devoid of transcription factor activity by point mutations at key amino acids of the recognition helix of the homeodomain [43, 44] (Caup^HD^* 1 and Caup^HD^* 2, Fig 6A). These mutant proteins were apparently less effective than wild-type Caup in restricting wing disc growth (Fig 6B, 6C and 6F). However, they were expressed at very reduced levels and showed both cytosolic and nuclear accumulation (Fig 6G–6I’ and S7G Fig), which could account for their low effect. Indeed, when expression was increased (flies raised at 29°C), they strongly reduced wing size (Fig 6D–6F). As expected, and even upon enforced expression, Caup^HD^* 1 or Caup^HD^* 2 were unable to repress *fng* expression and to prevent eye formation, although they notably reduced eye size (Fig 6M and 6M’ and S7J Fig). In S2 cells, Caup^HD^*1 and Caup^HD^*2 co-immunoprecipitated with CycE similarly to wild-type Caup (Fig 6J and S7K Fig). Moreover, the ability of Caup^HD^*2 to reduce wing size was abolished when this protein was additionally mutated at the IRO·box (Fig 6F). Hence these data support the binding of Caup to CycE-containing complexes, mainly through the IRO·box, as the main molecular mechanism for its function in the control of the cell cycle.

### *Iro* proteins regulate growth in *Drosophila* tumour models

Our results demonstrated the ability of Iro proteins to restrict cell cycle progression during normal development. Next, we addressed whether they were able to do so in *Drosophila* tumour-like models.

**Fig 7:**
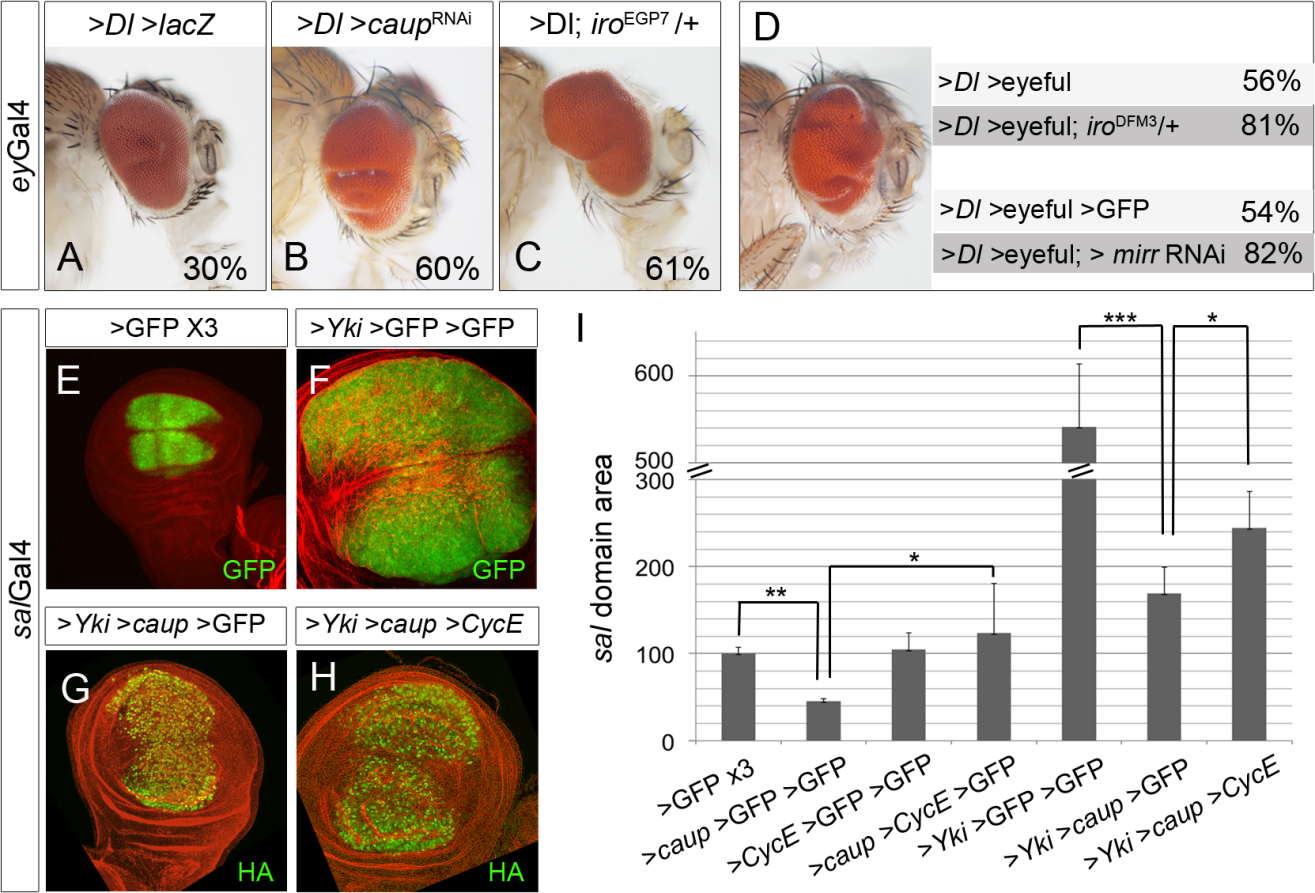
The levels of Iro proteins modulate tumour-like growth. (A-C) Depletion of Iro proteins enhances eye growth in the sensitized background *eyGal4>Dl>LacZ* (note the enlarged and folded eyes in B, C, compare with A). Representative eyes are shown, along with the percentage of enlarged eyes for each genotype (average from two independent experiments, n>80 each). Flies were raised at 29°C. (D) Reduction of *iro* function (*iro* ^DFM3^ */+*, *or mirr* depletion) enhances tumour-like growth in the >*Dl* >*eyeful* tumour model. (Left) Representative enlarged tumourous eye. (Right) Percentage of enlarged eyes in flies of the indicated genotypes (n>100, average value of three independent experiments). (E-I) Over-expression of *caup* reduces *yki*-induced overgrowth by CycE /Cdk2 inhibition. Compare the size of the *sal* domain (in green) in wing discs of the indicated genotypes. (G) Quantification of the area of the *sal* domain in third instar wing discs. Size domain was normalized to that of a *sal>*GFP*>*GFP*>*GFP wing discs (*p< 0.0001; **p<0.01). Discs are counterstained with Phalloidin (red).

Over-expression of the Notch ligand Delta (Dl) causes the development of slightly enlarged eyes (*eyGal4>Dl>lacZ* flies, Fig 7A) and provides a sensitized genetic background useful to identify genes affecting cell proliferation and tumorigenesis [45]. We tested whether reduced activity of any of the *Iro* genes affected the size of *eyGal4>Dl>lacZ* eyes. Indeed, while partial depletion of Caup on its own had no discernible effect on eye size (S8B Fig), it increased both the size and the number of eyes that showed severe folding (Fig 7A and 7B). Similar enhancement of this mutant phenotype was obtained by co-expressing *Dl* and RNAi constructs targeted to *ara* or *mirr* (S8A, S8C and S8D Fig) or in combination with *iro* ^EGP7^/+ (Fig 7C, 61% of the *eyGal4>Dl>iro* ^EGP7^/+ eyes were enlarged compared with 39% of the eyes in *ey*Gal4*>Dl* control flies).

Co-expression in the eye disc (driven by *ey*Gal4) of *Dl* and the epigenetic silencers *Pipsqueak* and *Lola* (referred to as >*Dl* >*eyeful* flies) induces the formation of tumour-like overgrowths in the eye [45]. Frequency of tumour formation was enhanced when >*Dl* >*eyeful* flies were in addition heterozygous for *iro* ^DFM3^ or depleted of Mirr (Fig 7D).

Thus, Iro depletion enhanced tumorous growth in the eye. Next we assayed whether, conversely, over-expression of *caup* reduced the overgrowth of the wing disc in another tumoral model. The Hippo pathway controls organ size in *Drosophila* and vertebrates by a coordinated regulation of proliferation and apoptosis and its dysfunction is frequently detected in human cancers [46]. Over-expression of the downstream component of the Hippo pathway *yorkie* (*yki*, *sal*Gal4*>yki*) increased the size of the territory where it is expressed (Fig 7E, 7F and 7I). We observed that co-expression of *caup* alleviated the overgrowth caused by *yki* (Fig 7G and 7I). One of the effects of *yki* over-expression is the activation of *cycE* transcription ([47], S6B Fig). Therefore, we hypothesized that the phenotypic suppression by Caup could be due to CycE/Cdk2 inhibition. Indeed, *cycE* co-expression partially reverted the effect of *caup* on *yki*-induced overgrowth (Fig 7E–7I). In sum, our data suggest a role of *Iro* genes as TSGs in *Drosophila*.

## Discussion

The identification of genes that control cell proliferation is paramount in developmental and cancer biology. The Iroquois proteins play multiple roles in regionalization and patterning during *Drosophila* development (reviewed in [12]). Here we show that they are also involved in the control of cell proliferation and, interestingly for homeodomain-containing proteins, they appear to do so by a non-transcriptional mechanism. This novel function of *Iro* genes would help developmental fields to attain their correct size and, if altered by *Iro* down regulation, could be a critical step for tumour progression.

We have analyzed *iro* hypomorphic and over-expression conditions and found that Iro proteins negatively control the G1-S transition of the cell cycle. *caup* over-expression impaired the activity of CycE/Cdk2 complex, while simultaneously increased the level of CycE protein. Still, CycE appears to be a limiting factor since its exogenous administration restores cell proliferation, while its reduction enhances it. The presence of Caup in CycE-containing protein complexes allow us to propose that this physical interaction inhibits CycE/Cdk2 activity thus slowing down cell proliferation. This hypothesis is supported by our observation that Caup^cyc*^ and Caup^IRO·box*^ mutant proteins show both impaired ability to co-immunoprecipitate with CycE and to restrict cell cycle progression. Although not experimentally demonstrated, we speculate that Caup may interact with CycE and Cdk2 containing complexes and inhibit their activity by preventing substrate recognition and/or stabilizing p21 binding. Further work is required to determine more precisely these molecular interactions. Since Caup^cyc^*^IRO·box^* still retains some ability to repress cell proliferation, we presume that either the functionality of these domains was not completely abolished by the mutations generated or the existence of additional unidentified interacting sites.

Although other homeobox proteins (and also some epigenetic regulators) have been shown to modulate the activity of cell cycle regulators by protein-protein interaction, many of them do it through transcriptional regulation [48–50]. We can rule out a transcriptional effect of Caup on cell cycle regulation since transcriptionally inactive Caup^HD^*1 and Caup^HD^*2 are still able to inhibit cell cycle progression.

Iro proteins play redundant roles in several developmental contexts [14, 15]. Here we show that the three of them are able to repress cell cycle progression when over-expressed and that this effect is abrogated by co-expression of *cycE*. The presence of putative Cyclin binding motives and the high conservation of the IRO·box in the Iro proteins [11] led us to propose that Ara and Mirr may also physically interact with CycE containing complexes. Since we found that the penetrance of the dorsal eye enlargement phenotype increases by reducing the overall amount of Iro proteins, we suggest that they may act in a redundant manner to modulate CycE/Cdk2 activity. Alternatively, the three Iro proteins may be functioning in a stoichiometric complex, this explaining why depletion of only one of them causes eye enlargement.

The present results suggest a novel role of Iro proteins as cell-autonomous regulators of the growth of the domains of the imaginal discs where they are expressed. Furthermore, our results fit to a current model that suggests that growth of territorial fields modulates the response of cells to morphogens (reviewed in [3]). In the eye discs, the ability of Decapentaplegic (Dpp) to induce retina differentiation is counteracted by Wg emanating from the anterior-most region of the discs (reviewed in [18] until the disc attains a size such that *dpp* expressing cells are beyond the range of action of Wg [7]. Accordingly, we suggest that the enhanced cell proliferation found in *iro* mutant discs, would enlarge the physical separation between Wg- and Dpp-expressing cells in the dorsal domain, thus increasing the efficiency of Dpp signalling and causing dorsal eye enlargement.

In analogy with this model for eye disc development, specification of the wing driven by Wg in the distal part of the wing disc is counteracted by the Vein morphogen, which spreads from the most proximal part of the wing disc (reviewed in [3]). In this scenario, reduction of the size of the distal wing disc by inhibition of cell proliferation prevents wing development (with the concomitant generation of a notum-like tissue, as shown in [8] and in this work), by facilitating the inhibition of Wg by Vein. Interestingly, Vein activates Iro gene expression in the notum region [38, 51] while Wg do so in the dorsal eye disc [14, 52–55]. Thus, we propose that *Iro* genes could provide a molecular mechanism that allow the ligands Vein (in the notum) and Wg (in the dorsal eye) to regulate the size of the morphogenetic field in which they operate.

Our results further suggest that a direct regulation of cell cycle progression by Iro/Irx proteins may be relevant for tumorigenesis. Thus, tumorous-like growth was observed in the eye imaginal discs when *iro* function was reduced in a sensitized genetic background (such as *ey>Dl* or *ey>Dl* >*eyeful* flies). Conversely, we show the ability of *caup* over-expression to counteract the overgrowth induced by Yki in imaginal discs, and that this is partially mediated by cycE/cdk2 inactivation. These data suggest a role of *Iro* genes as TSGs in *Drosophila* and agree with the association found between loss or reduced expression of members of *Irx* gene family and certain types of human cancer [20–23]. Note however that the role of *Iro*/*Irx* genes in tumorigenesis may be cell type-dependent since in some cases they appear to act as oncogenes [55 56]. Considering the presence of the IRO box [11] and of putative Cyclin-binding domains in Irx proteins (http://elm.eu.org), we hypothesize that some *Irx* mutations may contribute to cancer progression in vertebrates by increasing the activity of the CycE/Cdk2 complex and thus accelerating the G1-S transition, a key step frequently affected in cancer cells [57].

## Materials and Methods

### Site-directed mutagenesis of Caup

The following Caup mutations (*caup*-*mut*) were generated: *Caup* ^cyc^*, deletion of amino-acids 365 to 367 (RGL) of the Caup putative Cyclin-binding domain (RGLAP); *Caup* ^IRO·box^*, substitution of the only two positively charged amino acids of the IRO·box, Lysine 459 and Lysine 461 [11] to Ala; Caup^HD^*1, substitution of homeodomain Arginine 282 and Arginine 283 to Alanine [43] and *Caup* ^HD^*2, substitution of homeodomain Asparagine 279 to Alanine [44]. Mutants were obtained by site-directed mutagenesis (Quick-Change system, Stratagene) of wild-type *caup* cDNA [9] or *caup*-*mut* cDNA (this work) with the primers indicated in Supplemental Experimental Procedures.

### Over-expression experiments

Larvae expressing UAS-transgenes driven by *sal*Gal4; MD638Gal4 or *ey*Gal4 were raised at 25°C unless otherwise indicated. To increase the penetrance of the dorsally enlarged eye phenotype, *eyGal4; 2x UAS-mirr RNAi* larvae (Fig 1F) were raised at 29°C. To avoid the embryonic lethality associated with *caup* over-expression driven by *ap* and *hh* Gal4, we combined these lines with a *tub*Gal80^ts^ transgene [30]. Below 29°C, Gal80 inhibits Gal4 activity. Gal4 line; UAS-*iro* gene/*tub*Gal80^ts^ larvae were raised at 17°C, and transferred to 29°C 16 hours prior to dissection. In all experiments, the number of UAS genes was kept constant to avoid differences due to Gal4 titration. UAS-*caup*-HA and UAS-caup*-HA transgenic flies were obtained by the site-specific integration system at the 51D cytogenetic position [58] to get similar expression levels.

### Flow cytometry analysis

50 wing discs were dissected from *iro* ^DFM3^/*iro*Gal4, UAS-GFP larvae at 100–120h after egg laying. FACS analysis was done according to [28]. Cells were sorted by GFP expression using FACSCVantage SE (BD Biosciences) and cell cycle profiles were determined by Hoescht flourescence using a FACSCalibur flow cytometer (Becton Dickinson). Data from five independent experiments were analyzed using the FlowJo software and Dean-Jett-Fox model.

### Cell transfection and co-immunoprecipitation

*Drosophila* S2 cells were cultured in Insect-XPRESS media (Lonza) supplemented with 7% fetal calf serum and transfected using Nucleofector Technology (Lonza), according to the manufacturer’s specifications. *caup*-HA [59] and *caup-mut*-HA (this work) were cloned downstream of the constitutive promoter of the *Drosophila* Actin 5C gene in the pAc5.1 B plasmid (Invitrogen). The full-length *cycE* ORF was amplified from DGRC cDNA clone LD22682 using the following primers: 5’GAATCCGGCCGTACAATTATG3’ and 5’TCTAGAGGGATTGCTTCTAC3’ and cloned in pAc5.1 A (Invitrogen). Transfected cells were cultured during 48 hours before obtaining cell lysates by standard procedures. Antibodies used in immunoprecipitations and immunoblots were mouse anti-V5 (Invitrogen), mouse anti-GFP (Roche) and rat anti-HA (Roche). Similar results were obtained in at least two independent experiments.

### Wing size, mitotic index and pixel intensity determination

Areas of the *sal* domain of wing imaginal discs (n = 10) and of wings from female flies (n = 10; mounted in lactic acid /ethanol, 6:5) and pixel intensity of CycE-expressing cells were measured with Adobe Photoshop CS4. The values of CycE pixel intensity for each wing disc correspond to the ratio between average pixel intensity at the *sal* territory and the average pixel intensity at the adjacent territory (n = 10). To calculate the relative mitotic index, the number of pH3 expressing cells per area was quantified with Adobe Photoshop CS4 and then normalized to the mitotic index in the same region in control discs (n = 10).

### Statistical analysis

Data are shown as arithmetic mean ± standard deviation (SD, indicated by error bars). The statistical difference between groups of data was examined by Student’s t-test. p<0.05 was considered statistically significant.

## Supporting Information

S1 Fig Molecular and phenotypic analysis of *iro* mutations. (A) Scheme of the Iro-Complex showing in parenthesis the genomic regions deleted in the indicated *iro* deficiencies (*Df*). Arrows below the names of the *Iro* genes indicate their exon-intron structure. The homeodomain-encoding exons are shown in red. *Df(3L)iro* ^*EGP7*^ (*iro* ^*EGP7*^) is embryonic lethal, while *Df(3L)iro* ^*EGP1*^ (*iro* ^*EGP1*^) is fully viable. (B-F) Pattern of expression of the indicated *Iro* genes in third instar wild-type (WT, B, C) and *iro* ^EGP1^ */ iro* ^DFM3^ (D-F) eye discs (B, immunostaining; C-F, *in situ* hybridization). Hindsight accumulation (Hnt, green) in B labels the photoreceptor nuclei. The white arrow in B points at the morphogenetic furrow, an indentation of the disc epithelium that moves from posterior to anterior across the disc leaving differentiating ommatida in its wake [24]. (G- H´) Histological tangential sections of adult retinas of the indicated genotypes. (G´, H´) Dorsal and ventral ommatidial chirality is represented by arrows (black and red respectively) in the enlarged histological section of a wild-type eye (G´) and in the schematic representation of the *iro* ^EGP1^/*iro* ^DFM3^ eye (H´). Yellow lines indicate the position of the equator. (I-L) Dorsal (I, K) and lateral (J, L) views of nota form flies of the indicated genotypes. Red arrows point at the notopleural suture lost in *iro* ^EGP1^ flies. (M-P’) Down-regulation of *Iro* gene expression causes apoptosis (activated Caspase 3 staining) in wings (M, O) and eye (N, P, P’) discs. *iro* ^EGP7^ clones are labelled by loss of GFP staining in P, three of them are outlined. (M, N) Discs were counterstained with Phalloidin (red).
(TIF)

S2 Fig (A-C’) Analysis of wing disc cell size. The indicated UAS-trangenes were expressed during 16h prior to larvae dissection at late third instar stage. Transient over-expression of *caup* (B, b’) does not noticeably affect the size of wing disc cells (compare with A, a’). Similar transient depletion of CycE (*CycE* RNAi driven by *en*Gal4) does not increase wing disc cell size (C, c’). The broken yellow line indicates the limit between anterior and *en*-expressing posterior compartment cells. anti- aPKC staining was used to mark cell contours. (D-L) Cell cycle arrest caused by *ara*, *caup* or *mirr* over-expression is suppressed by *CycE* co-expression. pH3 staining (white) of wing imaginal discs that express the indicated transgenes driven by *nub*Gal4 (D-I) or *ap*Gal4 (J-L). The domains of expression of the Gal4 lines are outlined in D and J. Quantification of the relative mitotic index +/- SD is shown at the low right angle. (M-M”) Cell cycle arrest caused by *caup* over-expression driven by *sal*Gal4.
(TIF)

S3 Fig Effect of the over-expression of *caup* and cell cycle regulators on wing and wing cell size. (A) *caup* over expression (*sal*Gal4 driver) reduces both the length (proximo-distal, P/D axis, 14% reduction) and the width (antero-posterior, A/P axis, 19% reduction) of wings. Data were normalized to those of control *sal*Gal4>GFP wings. (B- C’) Expression of *wg* (immunostaining) in wing discs of the indicated genotypes. (D-I) Representative wings of flies of the indicated genotypes. (J-L) High magnification views of the intervein region boxed in F from wings of the indicated genotypes. Similar images were used to count the number of cells (each one producing a trichome) per fixed area and to obtain the numerical data presented in Fig 3H.
(TIF)

S4 Fig The mutant phenotype associated to *iro* gene over-expression is not suppressed by inhibition of apoptosis. (A-D) Representative wings of the indicated genotypes and wing size quantification (I). (E- E’) Ectopic over-expression of *caup*-HA driven by *sal*Gal4 increases apoptosis (activated caspase 3 staining) especially in the central part of the *sal* domain. (F, F’) Inhibition of apoptosis by DIAP1 co-expression. (G-H’) CycE accumulation in *caup* over expressing cells is not modified by inhibition of apoptosis, quantification in J (*** p<0.0005).
(TIF)

S5 Fig Modulation of the phenotypic effect of *caup* over-expression by *archipelago* (*ago*) activity. Representative wings of flies of the indicated genotypes (A-F) and wing size quantification (G).
(TIF)

S6 Fig (A-C) Analysis of *CycE* expression by *in situ* hybridization. Note the increase in *cycE* mRNA levels associated to *CycE* (A) and *yki* (B) over-expression and the decrease caused by *hippo* (*hpo*) over-expression (C, arrow points at the reduced *sal* domain in *sal*Gal4>*hippo* wing discs). (D-I) *caup* over expression does not affect *dap* expression. In wild type larvae, *dap* mRNA (D, E, *in situ* hybridization) and protein (G, H, immunostaining) accumulate at the morphogenetic furrow in eye imaginal discs (D, G) and show a generalized expression in the wing discs (E, H). *dap* expression is not modified by *caup* over-expression driven by *sal*Gal4 (F, I, the *sal* domain is boxed).
(TIF)

S7 Fig Sub-cellular localization, stability and activity of Caup mutant proteins (A-C´´). Nuclear localization of the indicated Caup proteins. Z-views of wing disc epithelium over-expressing the indicated transgenes driven by *sal*Gal4. Caup accumulation was determined by anti-HA staining, nuclei are labelled with DAPI and cell contours with Phalloidin. (D, E) Phenotypic effect of the over-expression of *caup* ^IRO·box^* (D) and *caup* ^cyc^* ^IRO·box^* (E). (F, G) Assay of the stability of the different Caup proteins. (*nub*Gal4, tubGal80^ts^ driver, see Materials and Methods). (Data shown as mean +/- SD). (H-J) Effect of the over-expression of different Caup proteins (*ey*Gal4 driver) on eye development. (K) Interaction of Caup^HD^*2 with CycE-containing complexes. Western blots of protein extracts from S2 cells expressing CycE-V5 and the indicated Caup-HA proteins, immunoprecipitated with anti-V5 antibody and probed with anti-HA. Black bars indicate the position of the 100 KDa protein marker.
(TIF)

S8 Fig Depletion of any Iro protein enhances eye overgrowth in *ey*Gal4*>Dl* flies. Individual RNAi-mediated reduction of Ara (A) or Caup (B) driven by *ey*Gal4, in otherwise wild-type flies, does not affect eye development. (C, D) Depletion of Ara (C) or Mirr (D) in the sensitized *ey*Gal4*>Dl* background enhances eye overgrowth. Representative eyes are shown, with indication of the average fraction of eyes displaying the shown phenotype in two independent experiments (n>80 each). Flies were raised at 29°C.
(TIF)

S1 Text *Drosophila* strains used in this study and supplemental experimental procedures. (PDF)
