# Supplementary figures and images for: The Homeodomain Iroquois Proteins Control Cell Cycle Progression and Regulate the Size of Developmental Fields
Source: PLoS Genet. 2015 Aug 25;11(8):e1005463. doi: 10.1371/journal.pgen.1005463 (PMC4549242; doi:10.1371/journal.pgen.1005463)

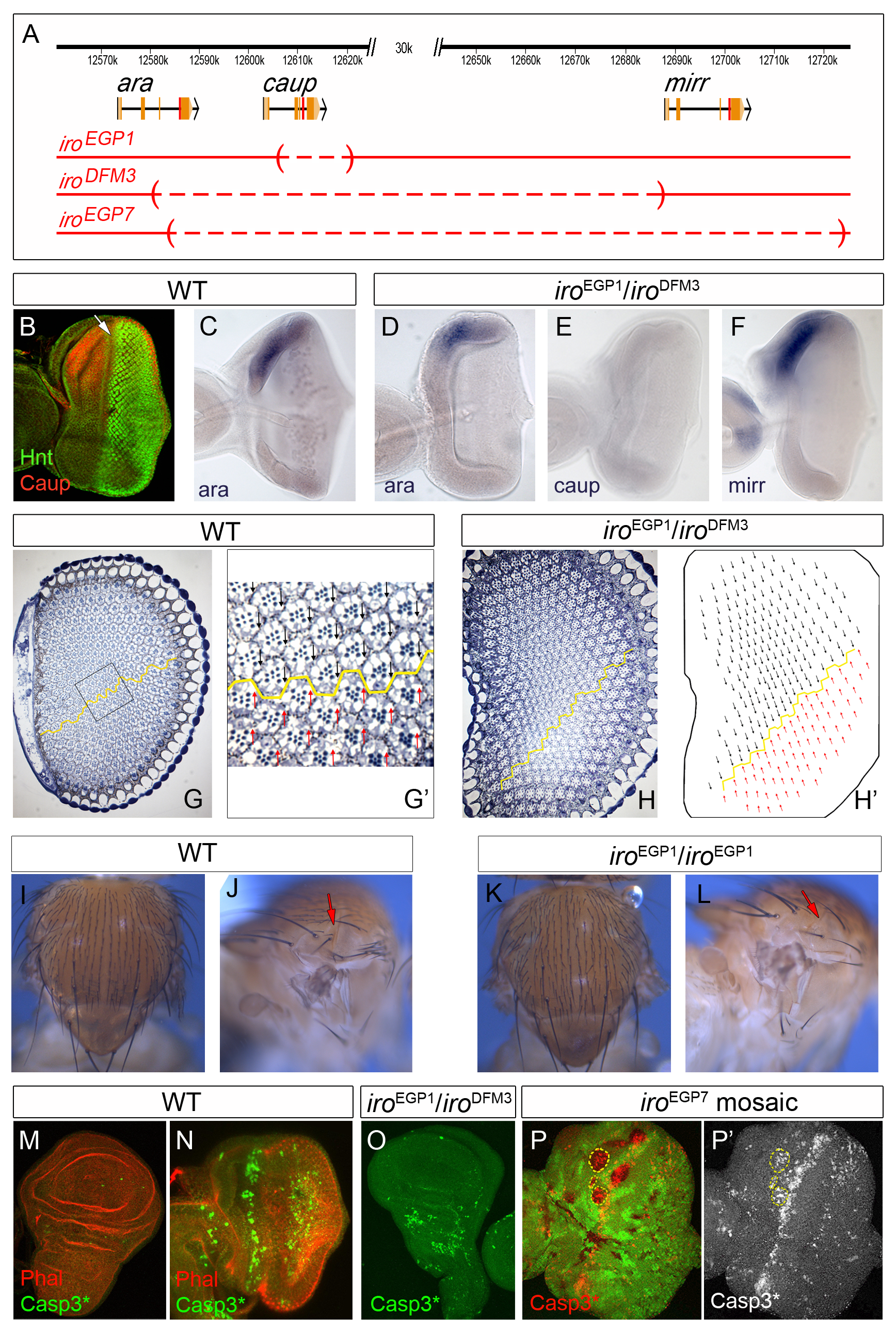

Supplement: S1 Fig — (A) Scheme of the Iro-Complex showing in parenthesis the genomic regions deleted in the indicated iro deficiencies (Df). Arrows below the names of the Iro genes indicate their exon-intron structure. The homeodomain-encoding exons are shown in red. Df(3L)iro EGP7 (iro EGP7) is embryonic lethal, while Df(3L)iro EGP1 (iro EGP1) is fully viable. (B-F) Pattern of expression of the indicated Iro genes in third instar wild-type (WT, B, C) and iro EGP1 / iro DFM3 (D-F) eye discs (B, immunostaining; C-F, in situ hybridization). Hindsight accumulation (Hnt, green) in B labels the photoreceptor nuclei. The white arrow in B points at the morphogenetic furrow, an indentation of the disc epithelium that moves from posterior to anterior across the disc leaving differentiating ommatida in its wake [24]. (G- H´) Histological tangential sections of adult retinas of the indicated genotypes. (G´, H´) Dorsal and ventral ommatidial chirality is represented by arrows (black and red respectively) in the enlarged histological section of a wild-type eye (G´) and in the schematic representation of the iro EGP1/iro DFM3 eye (H´). Yellow lines indicate the position of the equator. (I-L) Dorsal (I, K) and lateral (J, L) views of nota form flies of the indicated genotypes. Red arrows point at the notopleural suture lost in iro EGP1 flies. (M-P’) Down-regulation of Iro gene expression causes apoptosis (activated Caspase 3 staining) in wings (M, O) and eye (N, P, P’) discs. iro EGP7 clones are labelled by loss of GFP staining in P, three of them are outlined. (M, N) Discs were counterstained with Phalloidin (red). (TIF) [file pgen.1005463.s001.tif]

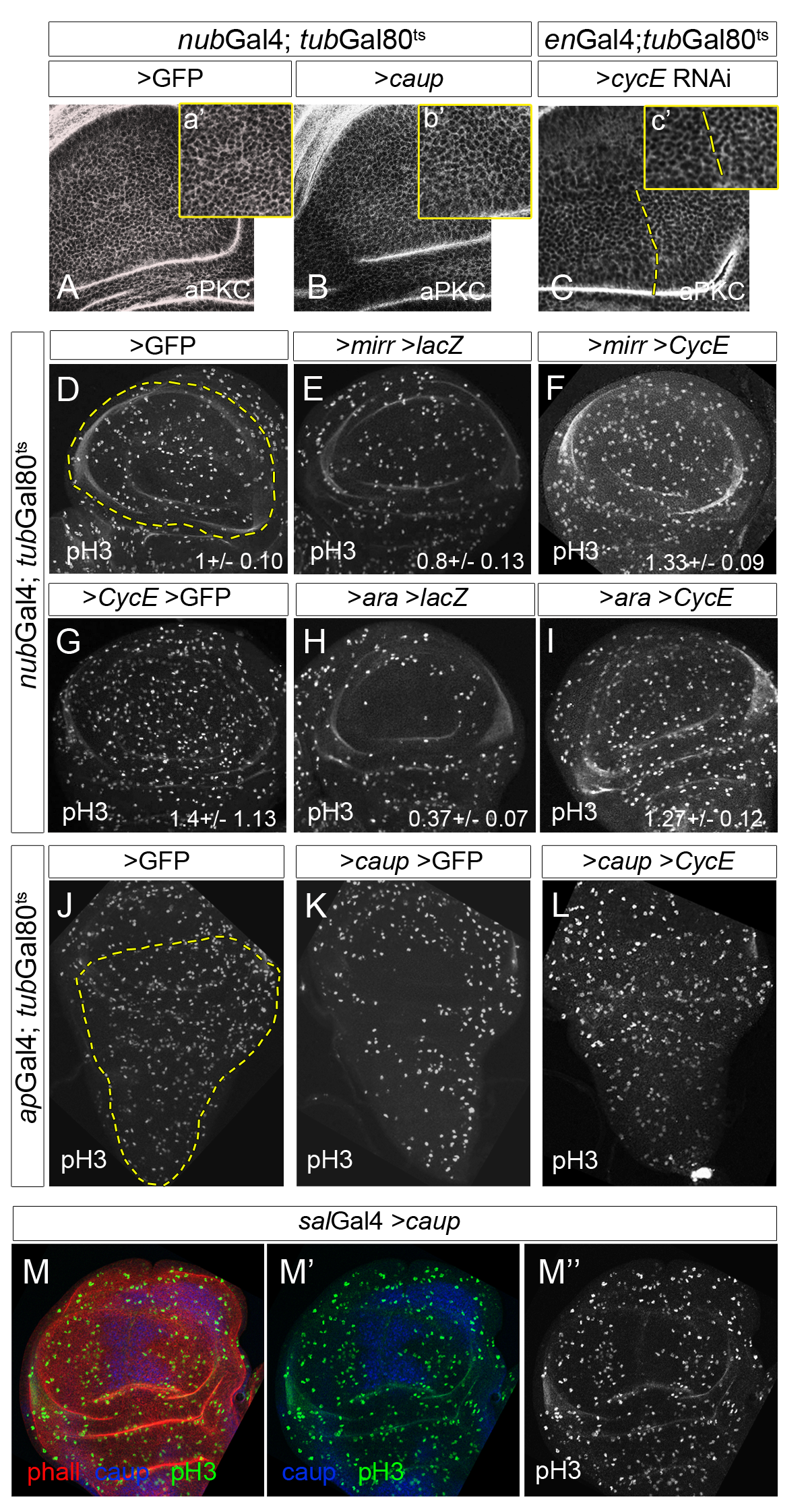

Supplement: S2 Fig — The indicated UAS-trangenes were expressed during 16h prior to larvae dissection at late third instar stage. Transient over-expression of caup (B, b’) does not noticeably affect the size of wing disc cells (compare with A, a’). Similar transient depletion of CycE (CycE RNAi driven by enGal4) does not increase wing disc cell size (C, c’). The broken yellow line indicates the limit between anterior and en-expressing posterior compartment cells. anti- aPKC staining was used to mark cell contours. (D-L) Cell cycle arrest caused by ara, caup or mirr over-expression is suppressed by CycE co-expression. pH3 staining (white) of wing imaginal discs that express the indicated transgenes driven by nubGal4 (D-I) or apGal4 (J-L). The domains of expression of the Gal4 lines are outlined in D and J. Quantification of the relative mitotic index +/- SD is shown at the low right angle. (M-M”) Cell cycle arrest caused by caup over-expression driven by salGal4. (TIF) [file pgen.1005463.s002.tif]

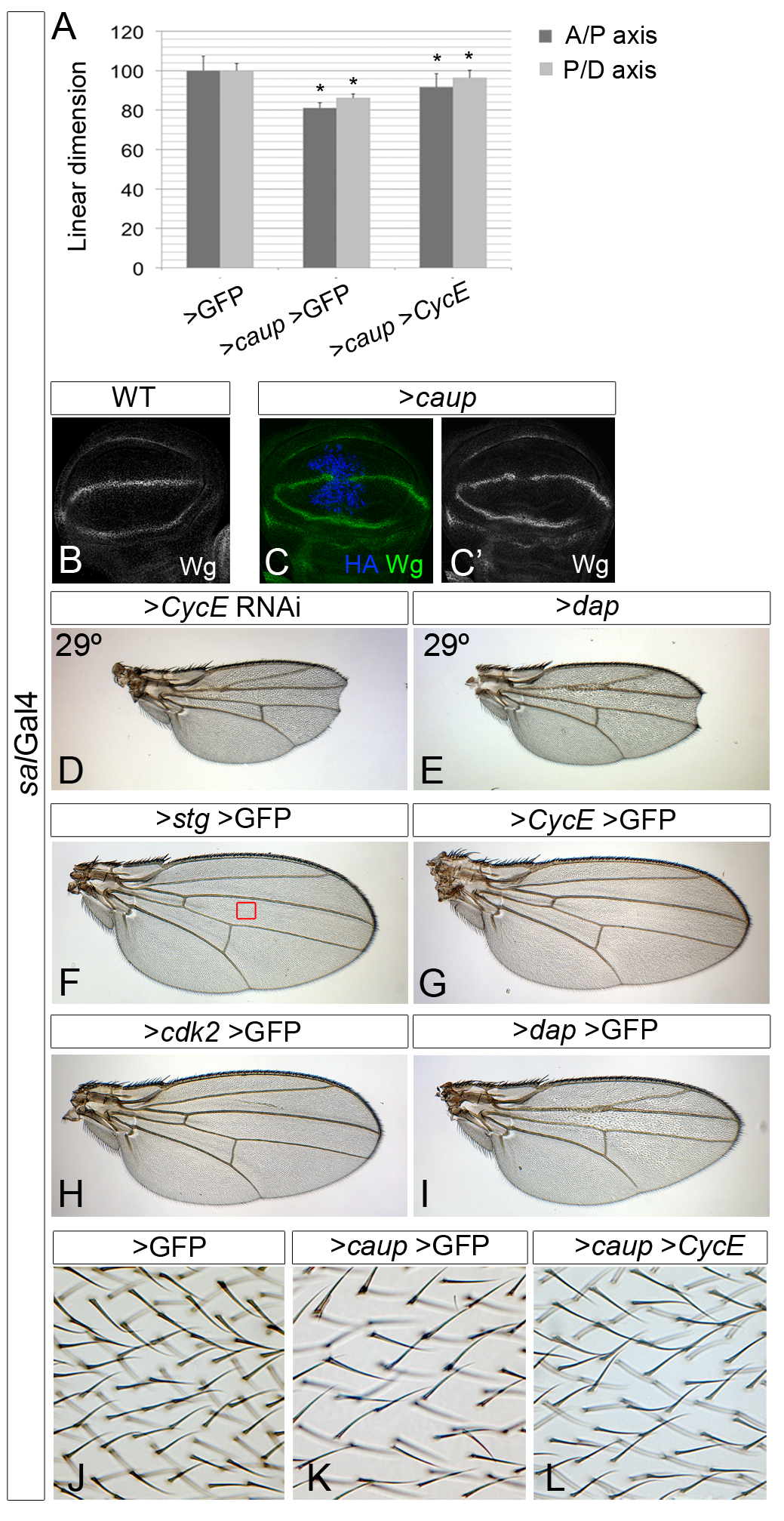

Supplement: S3 Fig — (A) caup over expression (salGal4 driver) reduces both the length (proximo-distal, P/D axis, 14% reduction) and the width (antero-posterior, A/P axis, 19% reduction) of wings. Data were normalized to those of control salGal4>GFP wings. (B- C’) Expression of wg (immunostaining) in wing discs of the indicated genotypes. (D-I) Representative wings of flies of the indicated genotypes. (J-L) High magnification views of the intervein region boxed in F from wings of the indicated genotypes. Similar images were used to count the number of cells (each one producing a trichome) per fixed area and to obtain the numerical data presented in Fig 3H. (TIF) [file pgen.1005463.s003.tif]

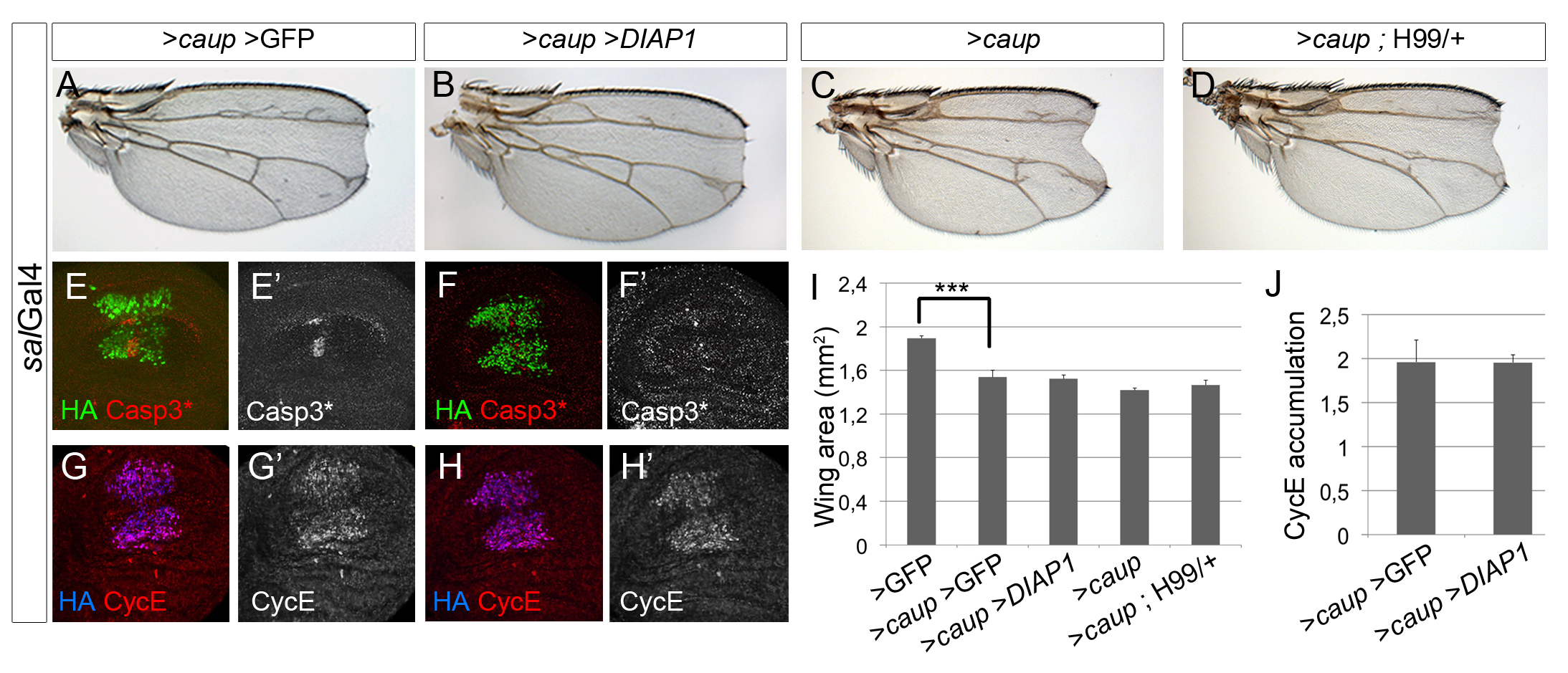

Supplement: S4 Fig — (A-D) Representative wings of the indicated genotypes and wing size quantification (I). (E- E’) Ectopic over-expression of caup-HA driven by salGal4 increases apoptosis (activated caspase 3 staining) especially in the central part of the sal domain. (F, F’) Inhibition of apoptosis by DIAP1 co-expression. (G-H’) CycE accumulation in caup over expressing cells is not modified by inhibition of apoptosis, quantification in J (*** p<0.0005). (TIF) [file pgen.1005463.s004.tif]

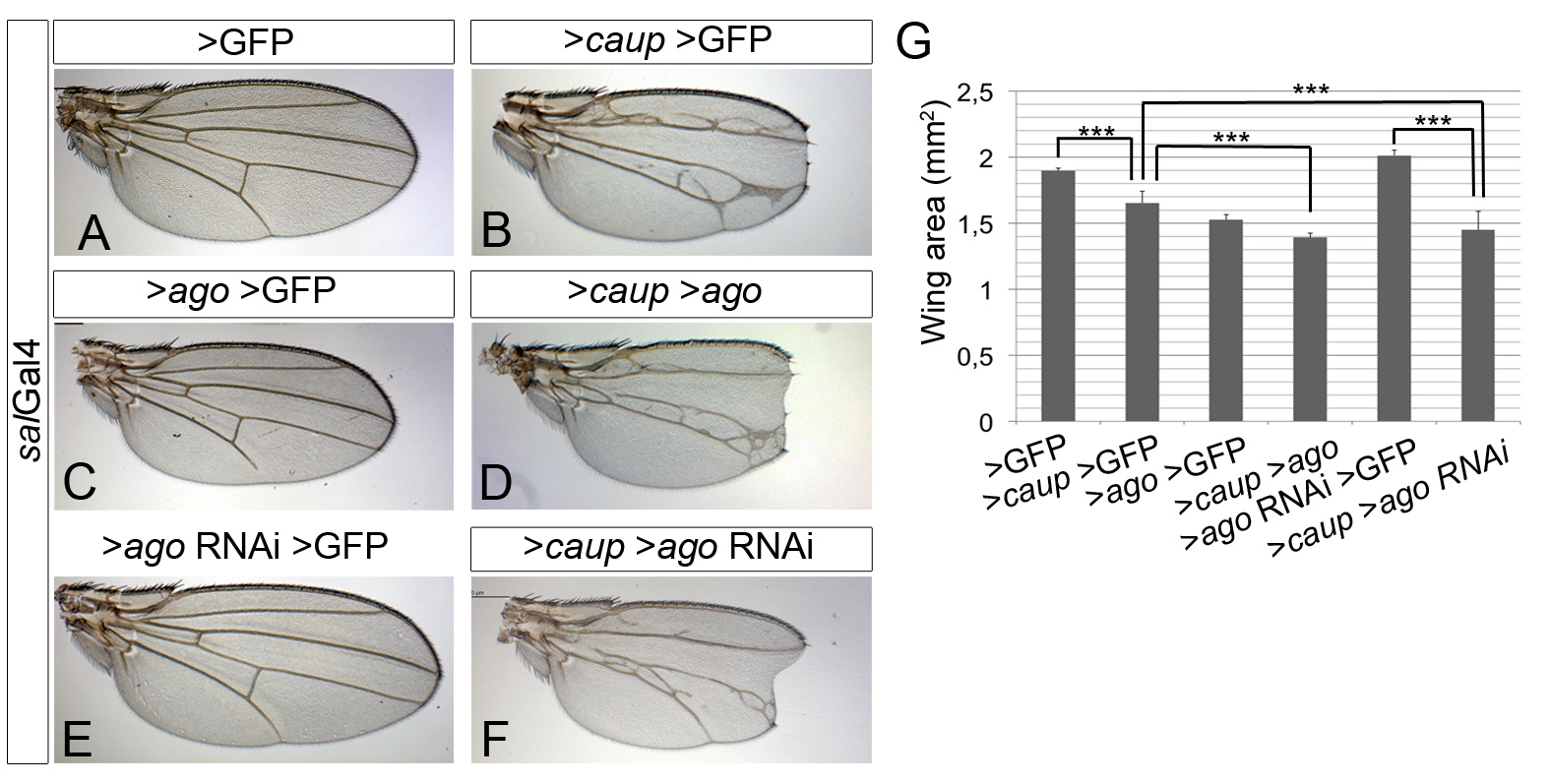

Supplement: S5 Fig — Representative wings of flies of the indicated genotypes (A-F) and wing size quantification (G). (TIF) [file pgen.1005463.s005.tif]

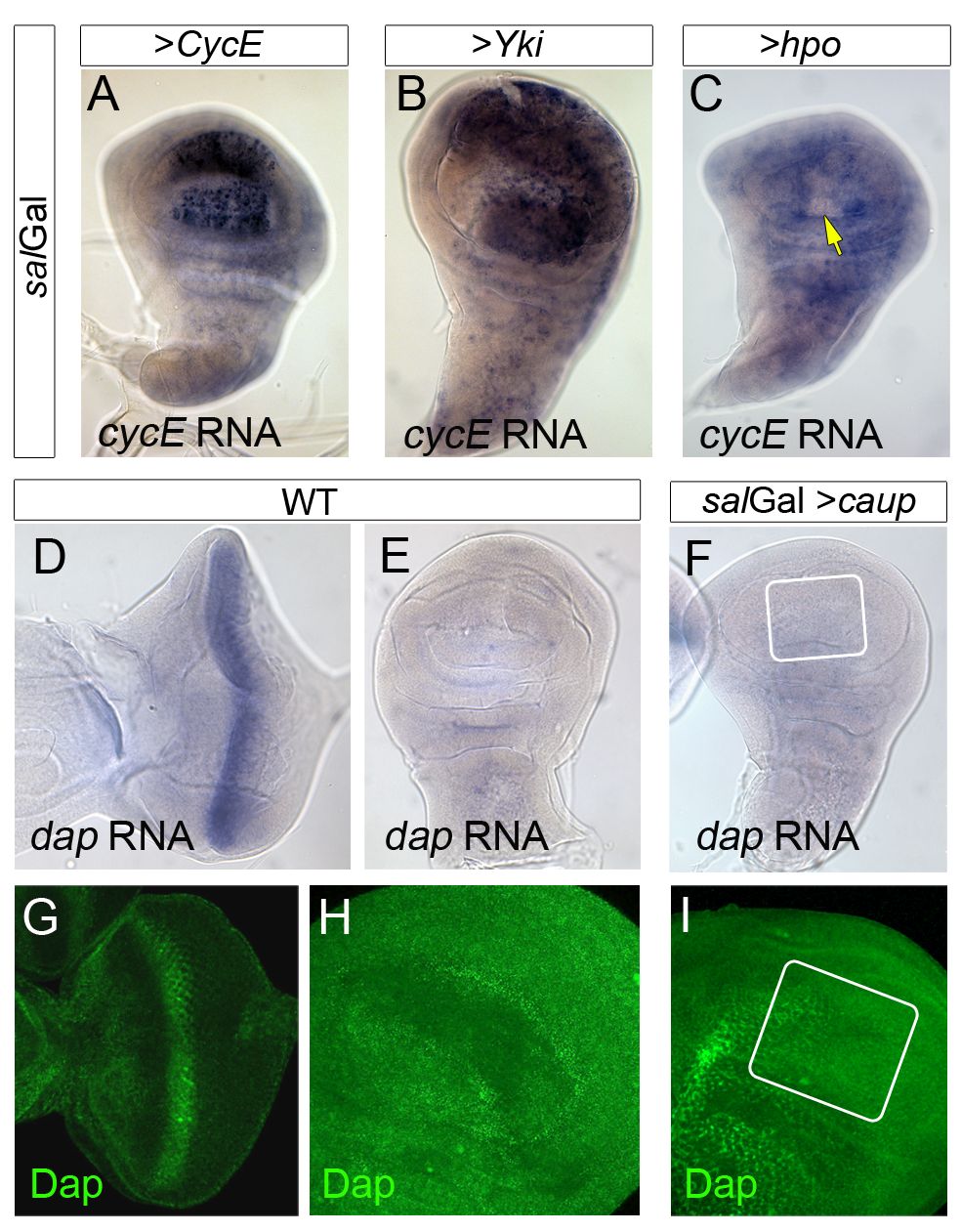

Supplement: S6 Fig — Note the increase in cycE mRNA levels associated to CycE (A) and yki (B) over-expression and the decrease caused by hippo (hpo) over-expression (C, arrow points at the reduced sal domain in salGal4>hippo wing discs). (D-I) caup over expression does not affect dap expression. In wild type larvae, dap mRNA (D, E, in situ hybridization) and protein (G, H, immunostaining) accumulate at the morphogenetic furrow in eye imaginal discs (D, G) and show a generalized expression in the wing discs (E, H). dap expression is not modified by caup over-expression driven by salGal4 (F, I, the sal domain is boxed). (TIF) [file pgen.1005463.s006.tif]

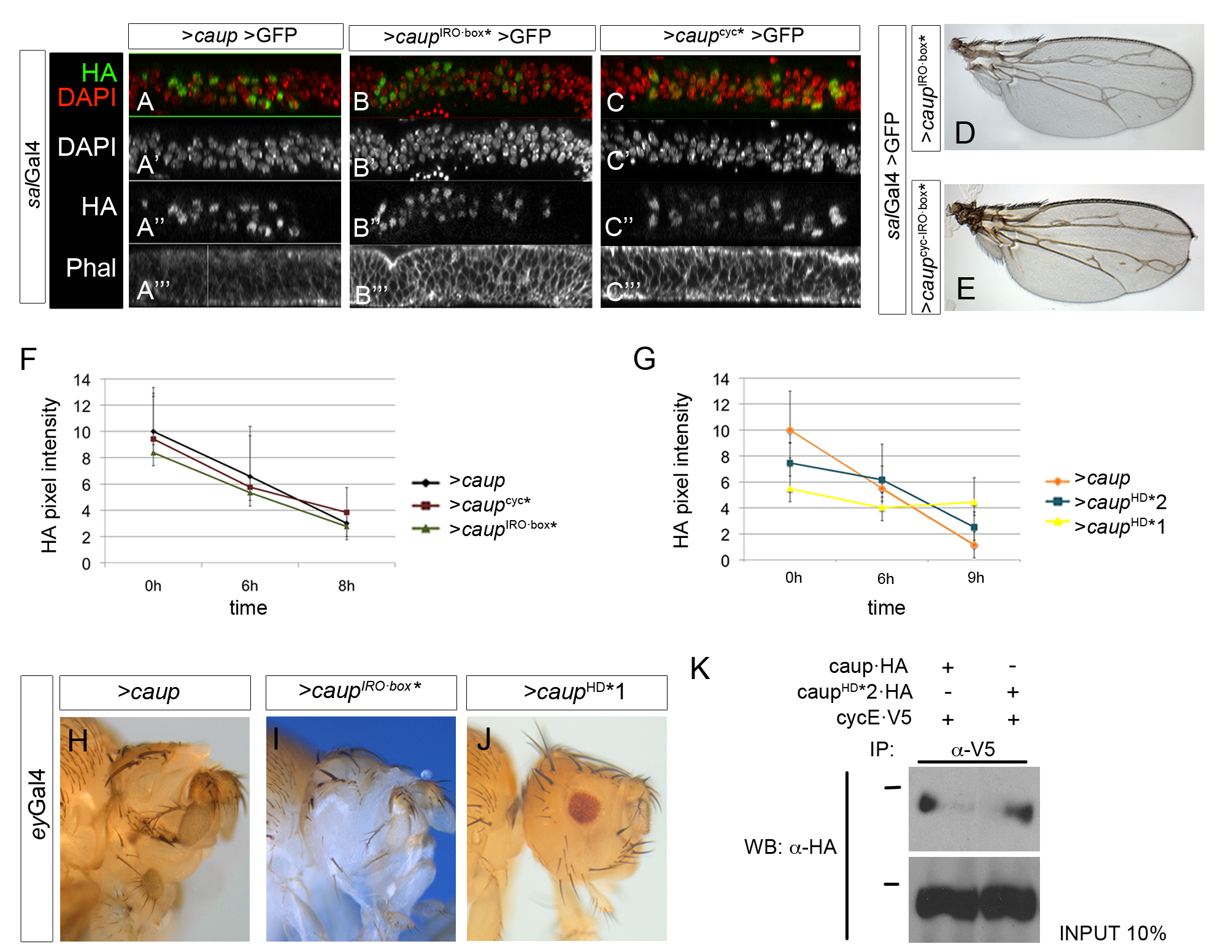

Supplement: S7 Fig — Nuclear localization of the indicated Caup proteins. Z-views of wing disc epithelium over-expressing the indicated transgenes driven by salGal4. Caup accumulation was determined by anti-HA staining, nuclei are labelled with DAPI and cell contours with Phalloidin. (D, E) Phenotypic effect of the over-expression of caup IRO·box* (D) and caup cyc* IRO·box* (E). (F, G) Assay of the stability of the different Caup proteins. (nubGal4, tubGal80ts driver, see Materials and Methods). (Data shown as mean +/- SD). (H-J) Effect of the over-expression of different Caup proteins (eyGal4 driver) on eye development. (K) Interaction of CaupHD*2 with CycE-containing complexes. Western blots of protein extracts from S2 cells expressing CycE-V5 and the indicated Caup-HA proteins, immunoprecipitated with anti-V5 antibody and probed with anti-HA. Black bars indicate the position of the 100 KDa protein marker. (TIF) [file pgen.1005463.s007.tif]

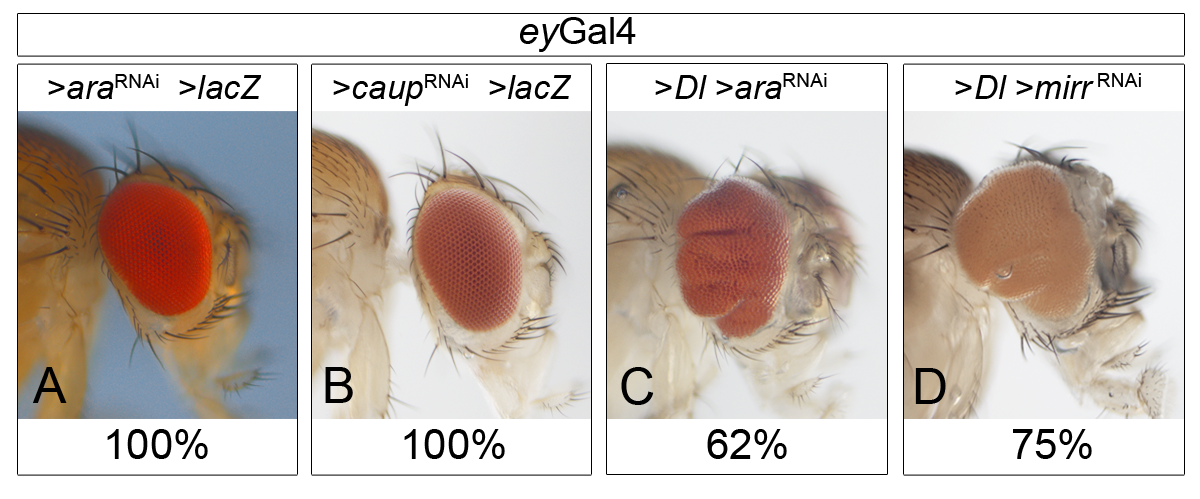

Supplement: S8 Fig — Individual RNAi-mediated reduction of Ara (A) or Caup (B) driven by eyGal4, in otherwise wild-type flies, does not affect eye development. (C, D) Depletion of Ara (C) or Mirr (D) in the sensitized eyGal4>Dl background enhances eye overgrowth. Representative eyes are shown, with indication of the average fraction of eyes displaying the shown phenotype in two independent experiments (n>80 each). Flies were raised at 29°C. (TIF) [file pgen.1005463.s008.tif]
