# Supplementary material for: The Homeodomain Iroquois Proteins Control Cell Cycle Progression and Regulate the Size of Developmental Fields
Source: PLoS Genet. 2015 Aug 25;11(8):e1005463. doi: 10.1371/journal.pgen.1005463 (PMC4549242; doi:10.1371/journal.pgen.1005463)
Supplement: S1 Text — (PDF) [file pgen.1005463.s009.pdf]

Supplemental Experimental Procedures

***Drosophila* strains**

Drosophila melanogaster OregonR and *y w* were used as wild-type strains. Stocks MD638Gal4, *ap*^{MD544}Gal4, *en*Gal4, *ey*Gal4, *hh*Gal4, *iro*Gal4, *nub*Gal4, *sal*^{PEv}Gal4 (named *sal*Gal4), UAS-*ara,* UAS-*Cdk2*, UAS-*cyclin E*, UAS-*cyclin A*, UAS-*dap,* UAS-*mirr,* UAS-*stg,* UAS-*yki*, UAS-hippo, P{EP}ago^{EP1135}, UAS-RNAi ago (BS# 31501), UAS-RNAi *cycE* (BS# 29314) and Df(3L)H99/Tm6b are described in FlyBase (http://flybase.org). Stocks *ey*Gal4, GS88A8, UAS-*Dl* / CyO (*ey*>*Dl*>*eyeful*) and *ey*Gal4, UAS-*Dl* / CyO were obtained from M. Domínguez [1], and UAS-*caup* RNAi (#105705), UAS-*ara* RNAi (#101903) and UAS-*mirr* RNAi (#50134) from the Vienna Drosophila RNAi Center. *sal*Gal4 driver is expressed in the central wing pouch of the wing disc from early third instar until 4h of pupal development [2]. A viable recombinant stock *sal*Gal4, UAS-*caup*-HA was generated in this work.

A DNA fragment encoding three copies of the hemaglutinin antigen (HA) was cloned into the pUASTattB vector [3] to generate a pUASTattB-HA vector. To generate *UAS-caup-HA* and each of the UAS-*caup* mutants-HA (UAS-*caup**-*HA*) transgenic flies, full-length wild-type *caup* ORF [4] or *caup** ORF (this work) were cloned in the pUASTattB-HA vector. All constructs were confirmed by sequencing before injection and by PCR sequencing of DNA obtained from single transgenic flies.

iro^{EGP1} and *iro*^{EGP7} are *iro* deficiencies generated by the FLP-FRT recombination method [5, 6] using pairs of FRT-bearing *piggyBac* Exelixis insertion lines, namely: caup^{f03450} and CG32111^{f03764} to generate *iro*^{EGP1}, and ara^{e02801b} and f03222 for *iro*^{EGP7}. The extent of these deficiencies and that of *iro*^{DFM3} [7] is shown in S1A Fig.

Clonal analyses

Marked clones of *iro* mutant cells were generated by FLP-mediated mitotic recombination [8]. *y w hsFLP*$^{1.22}$; *ubi-nlsGFP FRT2A /iro*^{EGP7} *FRT2A* larvae were subjected to a 1 hour heat shock at 37ºC at 72 hours after egg laying (AEL). Clones of cells over-expressing *caup** were obtained according to [9] by incubating *y w hsFLP*$^{1.22}$; UAS-caup*/ Actin FRT y+ FRT Gal4 UAS-LacZ larvae (at 48-72 hours AEL) at 37ºC for 10 minutes.

Immunostaining and histology

Immunostaining, *in situ* hybridization with and *in situ* hybridization combined with 5-bromo-4-chloro-3-indolyl-β-D-galactopyranoside (X-gal) staining, were performed as described [10, 11]. Digoxigenin-labelled anti-sense *ara*, *caup, mirr*, *cycE, dap* and *fng* RNAs were obtained with the DIG RNA labelling kit (Roche). The following primary antibodies were used: rabbit anti-Phospho-histone H3 (pH3) (1:200, Upstate Boitechnology); rat anti-HA (1:1000, Roche); rabbit anti-CycE (1:50) and rabbit anti-aPKC (1:50) from Santa Cruz Biothecnology; mouse anti-mpm2 (1:200, Millipore); rabbit anti-cleaved-Caspase 3 (1:50, Cell Signalling Technology); mouse anti-Dacapo (1:4), mouse anti-Hindsight (Hnt, 1:25) and mouse anti-Wg (1:100) from Developmental Studies Hybridoma Bank, DSHB) and rat anti-Caup (1:200, [7]. 5-ethynyl-2'-deoxyuridine (EdU) incorporation, to label cells in the S phase of the cell cycle [12] was assayed with Click-iT EdU Alexa Fluor (Invitrogen). Imaginal discs were counterstained with TRITC-Phalloidin (Sigma) to visualize cortical F-Actin. Images were obtained with confocal microscope LSM510 META (Zeiss). In all cases, the figures correspond to maximum-intensity z-stacks, generated in FIJI (ImageJ), of series of confocal sections.

Histological sectioning of adult eyes was performed as in [11].

Analysis of stability Caup proteins

*nub*Gal4; *tub*Gal80^{ts}; UAS-*caup**-HA larvae were raised at 17ºC until the early third

instar. Then, they were transferred to 29ºC during 16h (to inactivate $Gal80^{ts}$ and allow Gal4 activity). Just before transferring them again to 17 ºC (to prevent Gal4 activity), time 0h wing discs were dissected. Additional imaginal discs were dissected after 6h, 8h or 9h of further development at 17ºC. At least, two different experiments were preformed and a total of 6-10 discs were analyzed for *caup-HA* expression at each time point.

Site directed mutagenesis of Caup

Wild-type *caup* cDNA [4]was used to generate *Caup*^{cyc*} , *Caup*$^{Iro\ box*}$, *Caup*^{HD*} 1, and *Caup*^{HD*} 2.

Caup^{cyc*} was obtained by deletion of amino-acids 365 to 367 (RGL) of the Caup putative Cyclin binding domain (RGLAP) with the following primers:

forward (5'CACAATCTGGTGGCCATGGCG-CCATATGCCACGCCC3') and

reverse (5'GGGCGTGGCATATGGCGCCATGGCCACC-AGATTGTG3').

Caup$^{IRO\ box*}$ was obtained by substitution of amino acids Lysine 459 and Lysine 461 to Alanine with the following primers:

forward (5'CCAGTTCCGGCGAGCGCGCCCGCGATCTGGAGCGTGG3') and

reverse (5'CCACGCTCCAGATCGCGGGCGCGCTCGCCGGAACTGG3').

Caup^{HD*}1 was obtained by substitution of Arginine 282 and Arginine 283 (corresponding to amino acids R54 and R55 of the homeodomain) to Alanine with the following primers:

forward: 5'ACGTGGTTTGCCAATGCACGTGCTGCGCTGAAAAAGGAGAACAAG3' and reverse: 5'CTTGTTCTCCTTTTTCAGCGCAGCACGTGCATTGGCAAACCACGT3'

Caup^{HD*}2 was obtained by substitution of Asparagine 279 (corresponding to amino acid N51 of the homeodomain) to Alanine with the following primers: forward: 5'CCAGTTCCGGCGAGCGCGCCCGCGATCTGGAGCGTGG3' and reverse: 5'CCACGCTCCAGATCGCGGGCGCGCTCGCCGGAACTGG3'

Caup$^{\mathrm{IRO\ box}*}$ was used to generate *Caup*$^{HD2\text{-}\mathrm{IRO\ box}*}$ by site directed mutagenesis with the same primers used to generate *Caup*$^{\mathrm{HD}*}$ 2.

Caup$^{Cyc^{*}\ -\ \mathrm{IRO\ box}*}$ was generated by ligating appropriate restriction fragments from *Caup*^{Cyc*} and *Caup*$^{\mathrm{IRO\ box}*}$.

Supplemental References

[1] Ferres-Marco D, Gutierrez-Garcia I, Vallejo DM, Bolivar J, Gutierrez-Avino FJ, Dominguez M. (2006) Epigenetic silencers and Notch collaborate to promote malignant tumours by Rb silencing. Nature 439: 430-436.

[2] Cruz C, Glavic A, Casado M, de Celis JF. (2009) A gain-of-function screen identifying genes required for growth and pattern formation of the Drosophila melanogaster wing. Genetics 183: 1005-1026.

[3] Bischof J, Maeda RK, Hediger M, Karch F, Basler K (2007). An optimized transgenesis system for Drosophila using germ-line-specific phiC31 integrases. Proc Natl Acad Sci U S A. 104: 3312-3317.

[4] Gomez-Skarmeta JL, Diez del Corral R, de la Calle-Mustienes E, Ferres-Marco D, Modolell J. (1996) Araucan and caupolican, two members of the novel iroquois complex, encode homeoproteins that control proneural and vein-forming genes. Cell 85: 95-105.

[5] Parks AL, Cook KR, Belvin M, Dompe NA, Fawcett R, Huppert K, et al. (2004) Systematic generation of high-resolution deletion coverage of the Drosophila melanogaster genome. Nat Genet 36: 288-292.

[6] Thibault ST, Singer MA, Miyazaki WY, Milash B, Dompe NA, Singh CM, et al. (2004) A complementary transposon tool kit for Drosophila melanogaster using P and piggyBac. Nat Genet 36: 283-287.

[7] Diez del Corral R, Aroca P, Gomez-Skarmeta JL, Cavodeassi F, Modolell J (1999) The Iroquois homeodomain proteins are required to specify body wall identity in Drosophila. Genes Dev 13: 1754-1761.

[8] Xu T, Rubin GM (1993). Analysis of genetic mosaics in developing and adult Drosophila tissues. Development. 117: 1223-1237.

[9] Ito K, Awano W, Suzuki K, Hiromi Y, Yamamoto D (1997) The Drosophila mushroom body is a quadruple structure of clonal units each of which contains a virtually identical set of neurones and glial cells. Development 124: 761-771.

[10] Cubas P, de Celis JF, Campuzano S, Modolell J (1991) Proneural clusters of achaete-scute expression and the generation of sensory organs in the Drosophila imaginal wing disc. Genes Dev 5: 996-1008.

[11] Cavodeassi F, Diez Del Corral R, Campuzano S, Dominguez M (1999) Compartments and organising boundaries in the Drosophila eye: the role of the homeodomain Iroquois proteins. Development. 126: 4933-4942.

[12] Salic A, Mitchison TJ (2008) A chemical method for fast and sensitive detection of DNA synthesis in vivo. Proc Natl Acad Sci U S A. 105: 2415-2420.
